# RRT*-Guided Dual-Layer PPO Robust Control and Planning for Autonomous Bicycles in Rugged and Constrained Terrain

**DOI:** 10.3390/s26165087

**Published:** 2026-08-11

**Authors:** Rongjie Huang, Xiai Chen, Hang Deng, Jiongkun Yang

**Affiliations:** School of Mechanical and Electrical Engineering, China Jiliang University, No. 258 Xueyuan Street, Hangzhou 310018, China; p24010854039@cjlu.edu.cn (R.H.); dengh777@163.com (H.D.); s23010811031@cjlu.edu.cn (J.Y.)

**Keywords:** autonomous bicycle, deep reinforcement learning, hierarchical control architecture, global path planning, underactuated systems, unstructured environments

## Abstract

This paper addresses the problem that autonomous bicycles struggle to achieve precise obstacle avoidance and robust dynamic balance at the same time in rugged terrain and narrow constrained spaces. A hybrid hierarchical control architecture that integrates Rapidly-exploring Random Trees (RRT*) with a dual-layer Proximal Policy Optimization (PPO) scheme is proposed, referred to hereafter as the RRT*-2LPPO architecture. In the global planning layer, RRT* combined with cubic B-spline smoothing generates $C^{2}$-continuous reference trajectories. In the local control layer, decision-making and execution are hierarchically coupled through a dual-layer cooperation scheme. The upper-layer PPO network incorporates LiDAR data and uses the vehicle body pitch angle to enhance rugged terrain perception for heading planning, while the lower-layer PPO network coordinates the momentum wheel and the steering mechanism to maintain vehicle stability. Validation across various challenging scenarios demonstrates that the proposed framework achieves excellent robustness and consistently attains the highest navigation success rates, significantly outperforming traditional control methods and non-hierarchical architectures.

## 1. Introduction

With the rapid advancement of intelligent transportation systems and mobile robotics technology, the autonomous bicycle has emerged as a prominent lightweight mobile platform of growing interest in both academia and industry. Compared to four-wheeled vehicles, bicycles offer a significantly smaller footprint, superior maneuverability, and lower energy consumption, making them irreplaceable in resolving the “last-mile” delivery challenge, executing search-and-rescue missions in narrow spaces, and conducting security patrols in complex environments [1]. For instance, in post-earthquake rubble or congested urban alleyways, autonomous bicycles can traverse areas inaccessible to conventional vehicles, providing critical support for rescue operations and supply transportation.

However, the widespread deployment of autonomous bicycles depends fundamentally on their ability to navigate complex physical environments autonomously. As a typical underactuated, nonlinear, and nonholonomic constrained system [2], the bicycle is inherently unstable at low speeds, and its balance control and trajectory tracking are highly coupled. Traditional research in control science and engineering has primarily addressed the dynamic balance problem on flat surfaces; however, as application scenarios expand, enabling autonomous bicycles to adapt to rugged terrain such as stairs and steep slopes while simultaneously achieving precise obstacle avoidance and navigation within narrow, constrained spaces has become a critical challenge for improving environmental adaptability and intelligence. This requires not only a deep integration of mechanical dynamics modeling and control theory, but also the incorporation of advanced artificial intelligence algorithms to handle highly dynamic and uncertain environments.

Despite the substantial body of research on balance control and path tracking for autonomous bicycles, existing control strategies continue to face serious challenges when confronted with unstructured rugged terrain and geometrically constrained environments. From a dynamics perspective, the bicycle system is inherently underactuated, non-minimum-phase, and highly nonlinear. Conventional methods such as PID [3], LQR [4], and Sliding Mode Control (SMC) [5] largely rely on locally linearized dynamic models. When the vehicle traverses terrain such as stairs or steep slopes, where vertical loads fluctuate violently and ground contact points shift abruptly, model mismatch is significantly amplified, leading to severe degradation or even instability of the control system. On the other hand, while model-free learning-based methods such as deep reinforcement learning (DRL) [6] can effectively handle nonlinear system characteristics, end-to-end agents are often limited by sparse reward problems in long-range navigation tasks within narrow or maze-like environments. Without global guidance, they struggle to simultaneously balance high-frequency attitude stabilization and low-frequency path planning, frequently becoming trapped in local optima or repeatedly colliding in narrow passages. Furthermore, narrow terrain imposes strict geometric constraints on lateral vehicle motion, which naturally conflicts with the bicycle’s physical requirement to generate centrifugal force through steering maneuvers to maintain balance. Realizing precise obstacle avoidance and robust balance within an extremely limited feasible domain remains the key bottleneck constraining the all-terrain adaptability of autonomous bicycles.

To overcome the above limitations, this paper proposes the RRT*-2LPPO architecture, aiming to achieve robust control and intelligent planning for autonomous bicycles in complex three-dimensional terrain. The main contributions of this paper are as follows:

1. A global–local cooperative optimization architecture, RRT*-2LPPO, is proposed. The global planning layer uses the RRT* algorithm to generate a reference path, while the local control layer employs dual-layer PPO for obstacle avoidance and dynamic balance control. This design resolves the interference between global path guidance and local environmental response.
2. In the local control layer, an innovative dual-layer PPO algorithm is introduced. The upper-layer network incorporates LiDAR data and fuses the vehicle body pitch angle so that the vehicle can perceive slope changes, thereby achieving highly robust continuous climbing and descending on rugged terrain. The lower-layer network coordinates the momentum wheel and the steering mechanism to maintain high-frequency attitude stability, realizing high-robustness control of the autonomous bicycle in rugged terrain.
3. In the global planning layer, a two-stage global planning strategy combining the RRT* algorithm with cubic B-splines is designed. This strategy solves the problem that paths generated by standard RRT in narrow terrain have discontinuous curvature and cannot satisfy the bicycle’s nonholonomic constraints, making smooth tracking impossible. It also provides the local control layer with a reference trajectory, preventing local deadlocks and significantly improving task success rates and path smoothness in complex constrained scenarios.

It is worth emphasizing the specific algorithmic advance of the proposed framework beyond a mere integration of established components, as well as its distinction from closely related research lines. First, unlike hierarchical residual reinforcement learning [7,8,9], in which an RL policy learns residual corrections on top of a pre-designed base controller, the proposed framework does not rely on any hand-crafted base controller. Instead, the two PPO layers are assigned functionally distinct objectives with different observation and action spaces: the upper layer performs heading planning by fusing LiDAR data with the vehicle body pitch angle for rugged-terrain perception, while the lower layer coordinates the momentum wheel and the steering mechanism to maintain high-frequency attitude stability. This task-level decomposition, rather than a residual correction scheme, is what enables the bicycle to remain balanced while climbing stairs and steep slopes. Second, compared with existing autonomous-bicycle controllers, which typically address either balance control or path tracking in isolation, the proposed RRT*-2LPPO couples a globally optimal reference path with a dual-layer learned controller, so that precise obstacle avoidance and robust dynamic balance are achieved simultaneously in rugged and narrow constrained terrain.

The remainder of this paper is organized as follows. Section 2 reviews the related literature on autonomous bicycle motion control, path planning algorithms, and hierarchical control architectures. Section 3 details the proposed methodology, including bicycle dynamics modeling, the RRT*-based global path planning strategy, and the design of the dual-layer PPO controller. Section 4 presents the experimental setup, comparative results, and an in-depth quantitative and qualitative analysis of system performance in rugged terrain and narrow constrained spaces. Finally, Section 5 concludes the paper and outlines future research directions.

## 2. Related Work

### 2.1. Traditional Controller-Based Motion Control of Autonomous Bicycles

The autonomous bicycle system is a typical underactuated, non-minimum-phase, and unstable system with highly nonlinear dynamic characteristics [10,11]. Early control research primarily relied on linearized dynamic models. For example, Getz and Marsden [12] derived a feedback control method based on a bicycle model to stably track arbitrary smooth roll angle and rear-wheel speed trajectories. Chen et al. [13] used a fuzzy controller to control the roll angle of the bicycle, enabling it to track a given roll angle during stable circular motion. Bui et al. [14] employed a structure-specified $H_{\infty }$ loop-shaping controller for bicycle balance, which outperformed traditional proportional-derivative controllers and conventional $H_{\infty }$ loop-shaping controllers. Baquero-Suárez et al. [15] proposed a robust two-stage active disturbance rejection control method to maintain balance during bicycle riding.

LQR, as an optimal control method based on cost function optimization, has been applied by many researchers to the balance control of autonomous bicycles. Anjumol [16] proposed an LQR-based balance control strategy and additionally designed a linear trajectory tracking controller to achieve precise tracking based on trajectory parameters, with the feasibility of the proposed method verified through simulation. Owczarkowski [17] adopted a state-space representation based on the nonlinear Whipple model to derive the control method, and tested the effectiveness of the LQR approach for bicycle balance control. Wang et al. [18] first established the dynamic model of an autonomous bicycle using the Lagrangian method, and subsequently designed balance controllers for both stationary and moving conditions. Simulation results demonstrated that the stationary bicycle could maintain balance relying solely on the pendulum balancer, while the LQR controller performed well in the moving scenario.

Regarding trajectory tracking, Yavin [19] introduced the concept of path controllability; that is, for any two states and a finite time interval, if pedal and steering torques can be found to transition the bicycle from the initial state to the target state, the bicycle is deemed path-controllable. Based on a simplified dynamic model, the author derived control laws to achieve path planning and trajectory tracking between two given points. Yin and Yamakita [20] utilized passive velocity field control to achieve trajectory tracking for the bicycle robot. Based on the velocity field, this control method modifies the field’s form and introduces new control inputs to effectively suppress velocity changes caused by external forces, realizing precise control of the bicycle’s driving path. Bickford and Davison [21] investigated the problem of simultaneously stabilizing the bicycle and tracking a path, proposing a multi-loop control method that employs tilt torque and steering torque as control signals. This method demonstrated that the bicycle can asymptotically converge to a path with constant curvature. Shafiei and Emami [22] proposed a multi-loop control structure to track a predefined trajectory, where the inner loop controls the bicycle’s roll angle and the outer loop controls steering, thereby achieving both trajectory tracking and stability control. Wang et al. [23] proposed an EIC-structured controller featuring an internally and externally convertible structure. This structure can effectively describe the dynamic behavior of the robot during balancing and trajectory tracking, ensuring that the robot maintains balance and tracks the predefined trajectory under varying conditions.

### 2.2. Deep Reinforcement Learning-Based Motion Control of Autonomous Bicycles

In recent years, deep reinforcement learning (DRL) has achieved significant advances in solving high-dimensional continuous action space control problems by combining the perceptual capabilities of deep neural networks with the decision-making capabilities of reinforcement learning. Unlike traditional control, DRL adopts an “end-to-end” learning paradigm in which the agent directly learns the mapping from state space to action space through trial-and-error interaction with the environment, thus eliminating the dependence on precise dynamic models.

Briefly, reinforcement learning formulates the control problem as a Markov Decision Process (MDP): at each time step *t*, the agent observes the current state $s_{t}$, selects an action $a_{t}$ according to its policy $\pi_{\theta }\left(a_{t}|s_{t}\right)$, receives a scalar reward $r_{t}$ from the environment, and transitions to a new state $s_{t+1}$; the learning objective is to find a policy that maximizes the expected cumulative discounted reward. Among mainstream RL algorithms, Proximal Policy Optimization (PPO) [24] is an actor–critic method that updates the policy through a clipped surrogate objective, which restricts the deviation between the new and old policies at each update and thereby prevents destructive overly large policy updates. Owing to its training stability and ease of tuning, PPO is particularly well suited for continuous control tasks and is therefore adopted as the base learning algorithm in this work. The systematic instantiation of this RL formulation for the autonomous bicycle problem—including the state spaces, action spaces, and reward functions of the two PPO layers—is presented in Section 3.4.

In the area of autonomous bicycle balance control, Lakshmi and Manimozhi [25] used a Deep Deterministic Policy Gradient (DDPG)-based reinforcement learning method to address the self-balancing control problem of a motorcycle. They designed an agent that iteratively interacts with the environment to learn optimal control strategies by processing state information such as position and velocity and outputting motor torque commands. Shafiekhani et al. [26] proposed a combined fuzzy control and reinforcement learning approach for autonomous bicycle balance control, where the agent’s input is the system feedback interpreted as the last action taken in the previous state, and the generated signal is combined with error backpropagation for online adjustment of the fuzzy inference rules of the adaptive controller. Targeting the coupled control problem of balance and obstacle avoidance, Huo et al. [27] proposed an SAC algorithm with state prediction. By incorporating a Transformer-based time-series forecasting model, the agent is equipped with state prediction capabilities, achieving effective obstacle avoidance decision-making while maintaining vehicle balance.

In the aspect of path tracking, deep reinforcement learning achieves precise tracking of complex driving paths by directly learning the mapping from system states to control commands. Huo et al. [28] proposed an autonomous bicycle path tracking algorithm based on reinforcement learning, adopting an end-to-end approach to directly map system states to control commands. By introducing a curriculum learning strategy to design multiple training curricula from simple to complex, they effectively improved the algorithm’s convergence and achieved successful path tracking for the autonomous bicycle. Weyrer et al. [29] proposed a reinforcement learning training method that enables the bicycle to follow complex geometric paths such as circles and lanes, achieving path tracking while simultaneously accounting for the stable control of the vehicle.

### 2.3. Path Planning Algorithms for Mobile Robots in Complex Environments

Significant progress has been made in the development of path planning algorithms for mobile robots, emphasizing efficiency, adaptability, and safety. Various approaches have been proposed to address challenges such as dynamic obstacles, complex terrain, and multi-robot coordination. Zghair and Al-Araji [30] introduced a hybrid FAMCPSO algorithm that demonstrates a significant improvement in path length, reducing it by 43.3% and 25.5% compared to the RCD and A* algorithms respectively, highlighting its effectiveness in generating smoother and shorter routes in challenging environments. Similarly, Zheng et al. [31] proposed an artificial potential field-based particle swarm optimization algorithm (apfrPSO), which effectively addresses the limitations of traditional PSO algorithms, such as excessive path length and poor global search capability, thereby improving path quality. Nature-inspired metaheuristic algorithms have also been explored for their adaptability to dynamic environments. Xu et al. [32] provided a comprehensive classification of these algorithms, noting the shift from static to dynamic, high-dimensional, and multi-robot environments, which highlights the evolving complexity of path planning tasks.

In the domain of classical search algorithms, Li et al. [33] improved the traditional A* algorithm by integrating adaptive step adjustment and Bézier curves, reducing the number of turns by 50% and the path length by 3.62%. This integration addresses the issues of excessive turning points and prolonged computation time. Furthermore, Yang and Teng [34] enhanced the A* algorithm through improved search strategies and combined it with an improved Dynamic Window Approach (DWA), effectively reducing collision risks and improving path efficiency in random obstacle environments. The Dynamic Window Approach has been further optimized to improve local obstacle avoidance and path smoothness. Yang et al. [35] proposed an enhanced hybrid algorithm that fuses Ant Colony Optimization (ACO) with DWA, leveraging the global search capability of ACO and the local obstacle avoidance advantages of DWA. This approach also incorporates a novel path smoothing method to optimize the robot’s trajectory in complex environments. Building upon this, Yang et al. [36] developed a DWA-based distributed multi-robot navigation framework, introducing adaptive strategies and evaluation functions to improve path tracking accuracy and overall system robustness in unknown, dynamic environments.

Rapidly-exploring Random Tree (RRT), as a path planning technique based on incremental random sampling, rapidly expands toward unexplored regions by constructing a tree rooted at the start point within the state space. Its greatest advantage lies in eliminating the need for high-precision explicit modeling of the entire space, enabling it to extremely quickly explore a globally feasible path in high-dimensional complex spaces and under strong constraints (such as narrow passages), effectively avoiding local deadlocks. Shi et al. [37] proposed an improved RRT algorithm (GA_RRT) based on goal probability bias and cost functions, aiming to achieve path planning for dual-arm robots during obstacle avoidance. By introducing a bias strategy, this method effectively improves path search efficiency and obstacle avoidance performance, resolving the challenges in the coordinated motion of multi-arm robots. Dai et al. [38] proposed a novel potential-guided bidirectional RRT* algorithm combined with a direct connection strategy, which effectively avoids the tedious calculations of inverse kinematics and enhances the shortness and safety margin of the path. The application of this method in the joint space of redundant robots demonstrates superior performance, reducing the generation of invalid nodes. Recently, Sun et al. [39] proposed a phase search-enhanced Bi-RRT path planning algorithm for mobile robots, which improves the exploration efficiency, convergence speed, and path pruning of the bidirectional RRT framework, representing a recent advance in sampling-based planning.

Beyond classical and sampling-based planners, deep reinforcement learning has also been applied to path planning and obstacle avoidance. For example, Hua et al. [40] proposed an improved DDPG algorithm-based path planning method for unmanned surface vehicles, demonstrating the potential of deep reinforcement learning for continuous path-planning control and obstacle avoidance.

Overall, these studies collectively demonstrate a trend toward hybrid, adaptive, and intelligent algorithms that address the multifaceted challenges of path planning in complex environments, emphasizing the importance of combining global optimization, local obstacle avoidance, and real-time adaptability.

### 2.4. Hierarchical Control and Hybrid Architecture Research

End-to-end deep reinforcement learning methods, through direct interaction between the agent and the environment, can learn a mapping strategy from perception to decision-making directly from raw state information. This eliminates the dependence on precise dynamic models and demonstrates strong adaptive capabilities in specific scenarios. However, when faced with the multi-objective coupled task of balancing high-frequency attitude stabilization and low-frequency trajectory tracking for an autonomous bicycle, a single end-to-end strategy often struggles to simultaneously reconcile the high-frequency responsiveness required for dynamic balance with the low-frequency accuracy needed for path tracking. Furthermore, its inherent “black-box” nature leads to insufficient interpretability, making sample efficiency and training stability difficult to guarantee.

To address the limitations of the aforementioned end-to-end models, scholars have proposed hybrid architectures aiming to complement the robustness of traditional controllers with the adaptive capabilities of reinforcement learning. The core idea is to utilize an RL agent to compensate for the residual errors of a traditional controller or to handle unmodeled dynamics. Johannink et al. [7] systematically articulated the concept of Residual Reinforcement Learning (Residual RL) early on, laying the theoretical foundation for subsequent research. In the domain of autonomous bicycles, Zhu et al. [8] proposed a natural residual reinforcement learning framework that utilizes RL to optimize the output of a base controller to handle external disturbances. Subsequently, they further combined RL with adaptive Terminal Sliding Mode Control [41] to achieve high-precision adaptive control on curved surfaces in the presence of external disturbances. Additionally, Zhu et al. [42] proposed an online series-parallel combination controller design, fusing reinforcement learning with traditional control in a series-parallel structure: the parallel component utilizes DRL to compensate for the equilibrium point, while the serial component adjusts the sliding mode controller parameters online, thereby achieving stable path tracking on curved surfaces. For more complex terrain environments, Zhu et al. [43] explored the control problem of bicycle robots on rugged terrain based on deep reinforcement learning, validating the potential of hybrid strategies in suppressing terrain-induced oscillations.

However, when handling multi-objective conflicts, the aforementioned single-layer hybrid architectures remain confined to the same level of abstraction, making it difficult to effectively decouple control tasks operating at different time scales, and they lack the capability for active global path planning. Addressing the inability of these hybrid models to decouple multi-time-scale control tasks, researchers began adopting Hierarchical Control and Hierarchical Reinforcement Learning (HRL) to reduce system learning difficulty and coupling through structured policy decomposition. Bhatti et al. [44] validated the effectiveness of adaptive cascade control in a ball-riding robot, demonstrating that decoupling the balance loop (inner loop) from the position loop (outer loop) significantly enhances system performance. Inspired by this, Liu et al. [45] proposed a hierarchical coordinated trajectory tracking method, where an upper-level controller handles velocity constraints via reinforcement learning, and a lower-level controller achieves dynamic control using an LQR. This hierarchical integration of kinematic and dynamic control effectively reduced design complexity and enhanced system robustness. In recent advances, Huo et al. [9] (2025) proposed a path tracking method based on Hierarchical Residual RL, successfully resolving high-precision tracking issues for complex trajectories through a hierarchical design of upper-level planning and lower-level execution, representing the state of the art in this field.

Despite these advances, existing hierarchical architectures are mostly limited to optimization at the “local tracking” level; that is, the system typically assumes the global reference path is known, collision-free, and absolutely smooth. When confronted with extreme scenarios where narrow, constrained spaces are highly coupled with three-dimensional rugged terrain, this assumption completely fails, and simple tracking algorithms cannot resolve the feasibility problem of path search. Specifically, although Huo et al. [9] achieved hierarchical control, they lacked the capability for global path planning in environments with complex geometric constraints, making the system highly prone to local deadlocks in narrow dead ends. While Zhu et al. [43] addressed control on rugged terrain, their approach lacked refined obstacle avoidance strategies in narrow spaces, failing to achieve an effective unification of 3D terrain adaptation and global geometric navigation.

To solve these problems, this paper proposes and implements the RRT*-2LPPO architecture. In this framework, the global layer utilizes RRT* combined with cubic B-spline smoothing to generate collision-free reference trajectories satisfying $C^{2}$ continuity, fundamentally compensating for the lack of global planning capability in narrow spaces seen in existing hierarchical algorithms. At the local execution end, the hierarchical control layer achieves a hierarchical cooperation between decision-making and execution: the upper-layer PPO network introduces LiDAR data and utilizes the vehicle body pitch angle to enhance rugged terrain perception for heading planning; the lower-layer network is exclusively dedicated to coordinating the momentum wheel and steering mechanism to maintain high-frequency attitude stability under extreme disturbances.

## 3. Methodology

### 3.1. Overall System Architecture

**Figure 1:**
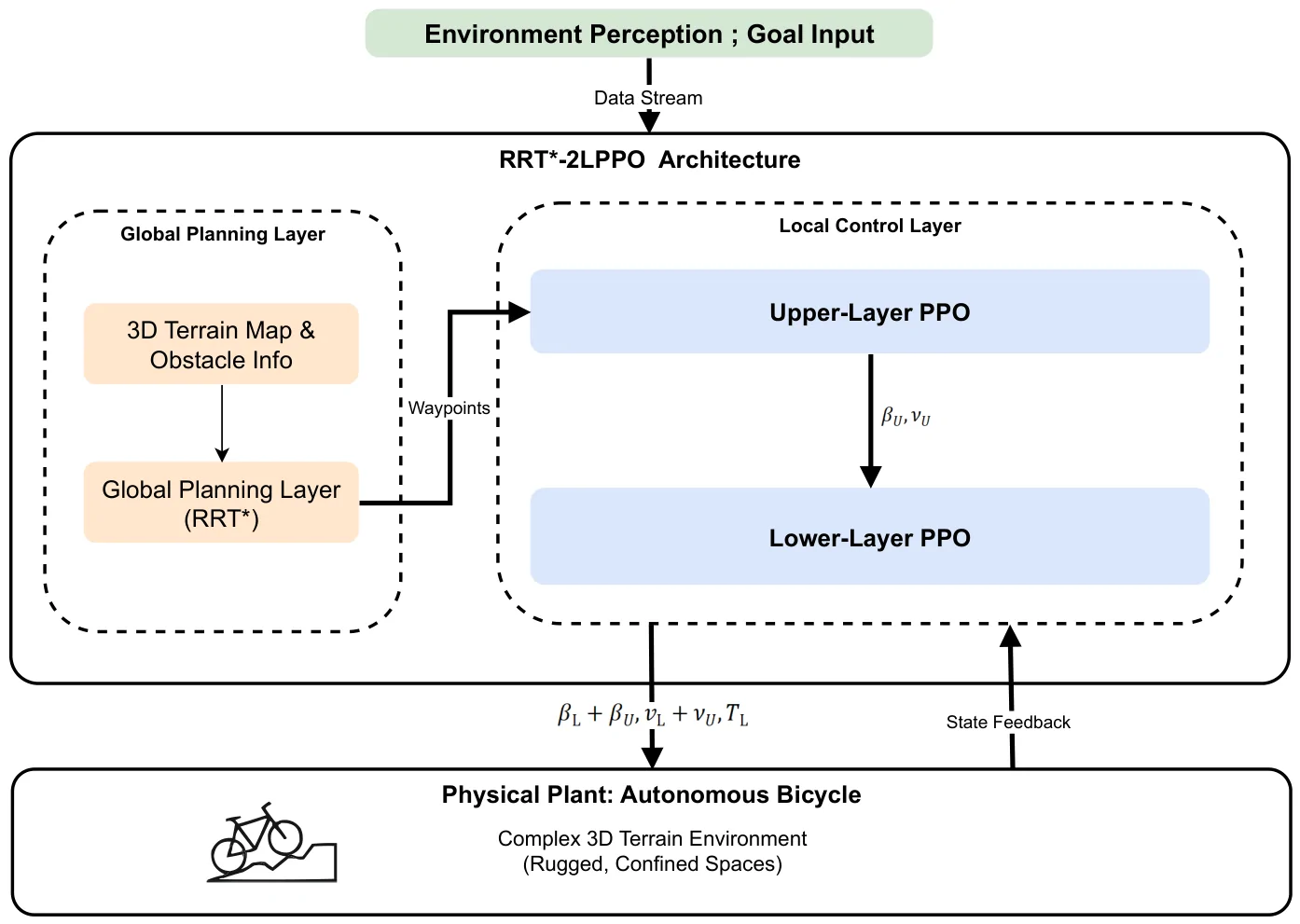
RRT*-2LPPO architecture diagram.

To address the control challenges faced by unmanned bicycles in rugged terrains and confined spaces, this paper proposes the RRT*-2LPPO architecture, as illustrated in Figure 1. The system aims to resolve three core conflicts inherent in navigating complex three-dimensional environments with underactuated systems: global navigation reachability, local trajectory tracking accuracy, and low-level dynamic balance robustness. The system consists of three core subsystems: the Global Planning Layer, the Local Control Layer, and the Physical Plant. The entire system operates through a closed-loop feedback mechanism. Information flows from top-level environmental perception data down to low-level torque outputs, while vehicle state feedback propagates inversely to the control layer, forming a complete Perception–Action Loop.

**Figure 2:**
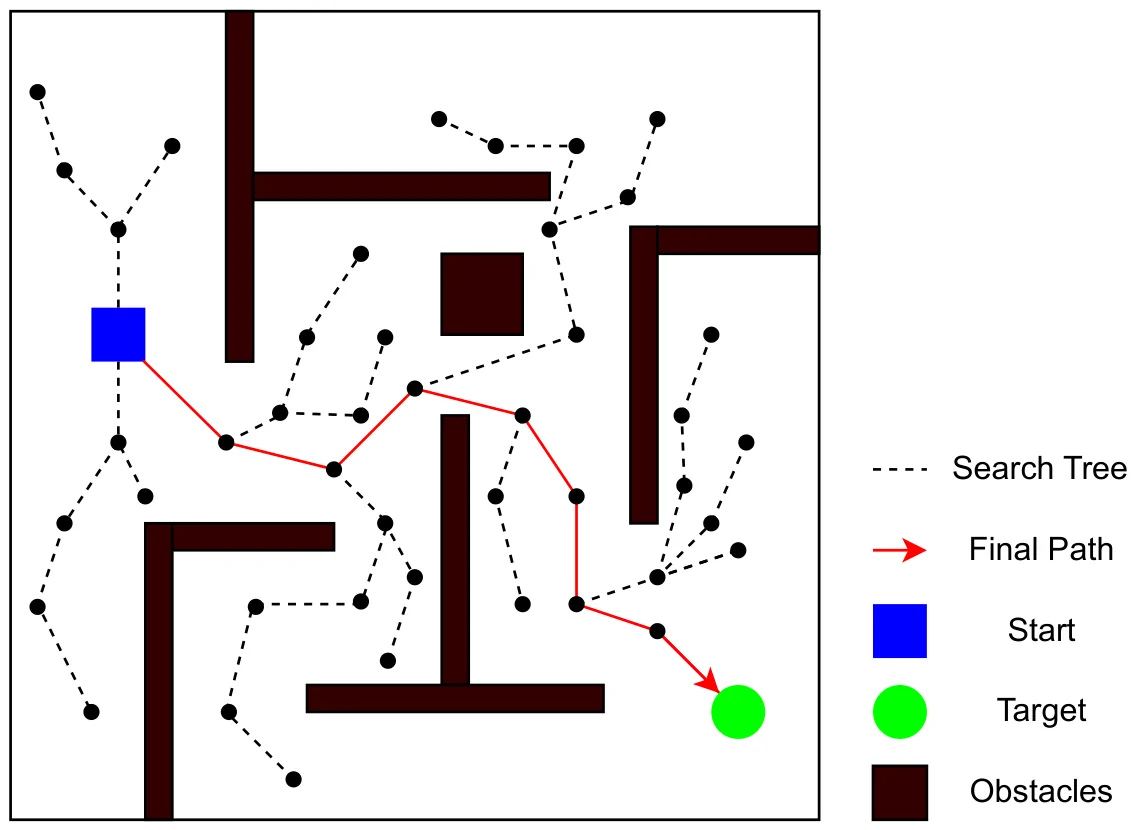
Schematic diagram of the RRT* algorithm.

Global Planning Layer. Located at the front of the architecture, this module transforms high-dimensional unstructured environmental information into low-dimensional geometric path constraints. Because narrow terrain is frequently accompanied by complex obstacle distributions and dead ends, traditional reactive obstacle avoidance algorithms are highly prone to falling into local minima when approaching the target. Therefore, this layer employs the RRT* [46] algorithm as the global planner to overcome geometric constraints. As illustrated in Figure 2, the RRT* algorithm starts from the start point and performs probabilistic sampling within the configuration space. Through iterative growth, it constructs a search tree (shown as dashed lines) that covers the free space. This search tree rapidly explores and bypasses the black rectangular obstacles. Once it reaches the target region, it backtracks to generate a collision-free geometric path connecting the start and goal points (the Final Path, shown as a red solid line). This path is logically discretized into a sequence of ordered waypoints, which are passed to the subsequent decision layer as macroscopic guidance data. This design effectively decouples “geometric feasibility search” from “dynamic control,” ensuring that the vehicle already possesses a theoretically feasible global pathway before entering complex terrain, and fundamentally preventing the risk of straying into deadlock regions.

Local Control Layer. This layer adopts a Dual-Layer PPO Network design. Through hierarchical functional division, it achieves a hierarchical cooperation between upper-level kinematic decision planning and lower-level dynamic execution.

Upper-Layer PPO. The upper-layer network receives sparse waypoints from the global planning layer, locally perceived obstacle information from the LiDAR, and the vehicle body pitch angle as state inputs. It achieves highly robust continuous climbing and descending on rugged terrain. Guided by waypoints, obstacle distributions, and terrain variations, it outputs desired compensation quantities $\left(\beta_{U},v_{U}\right)$ that satisfy the bicycle’s kinematic constraints, representing the desired steering angle compensation and velocity compensation, respectively. These compensation quantities are passed to the lower-layer PPO as reference guidance for terrain traversal.

Lower-Layer PPO. The core task of the lower-layer network is to solve the high-dimensional nonlinear vehicle coordination problem, especially coordinating the coupling relationship between the steering mechanism and the momentum wheel to maintain dynamic stability. The lower-layer network generates a set of base steering and velocity commands $\left(\beta_{L},v_{L}\right)$, and simultaneously receives the kinematic compensation quantities $\left(\beta_{U},v_{U}\right)$ from the upper layer. The two are superimposed to form the actually executed steering angle and velocity commands $\left(\beta_{L}+\beta_{U},\;v_{L}+v_{U}\right)$. In addition, the lower layer independently outputs the momentum wheel torque $T_{L}$, using the precession or reaction torque produced by the momentum wheel to assist balance. The three commands $\left(\beta_{L}+\beta_{U},\;v_{L}+v_{U},\;T_{L}\right)$ are merged into a single control signal output directly to the physical plant (steering motor, drive wheel motor, and momentum wheel motor), realizing complete action control of the bicycle.

Physical Plant. At the bottom of the system is the autonomous bicycle dynamics model in a complex three-dimensional rugged and confined terrain environment. The bicycle is a typical nonholonomically constrained and underactuated system. In rugged terrain, the contact points between wheels and ground continuously change, and the gravitational component varies in real time with the slope, posing great challenges to the robustness of the controller.

Regarding the information assumptions of the framework, the application scenario targeted in this work is a campus-like semi-enclosed environment, in which a two-dimensional map of the environment is known a priori and directly provided to the RRT* global planner. In contrast, the positions of local obstacles and the detailed ruggedness of the terrain surface are not assumed to be known: at execution time, the single-line LiDAR perceives local obstacles online, and the vehicle body pitch angle reflects the current slope state of the terrain being traversed, while the ability to traverse unknown rugged surfaces is acquired through extensive training in randomized rugged terrain. Therefore, no online three-dimensional reconstruction, SLAM system, or privileged simulator information is required at deployment.

### 3.2. Autonomous Bicycle Modeling Design

**Figure 3:**
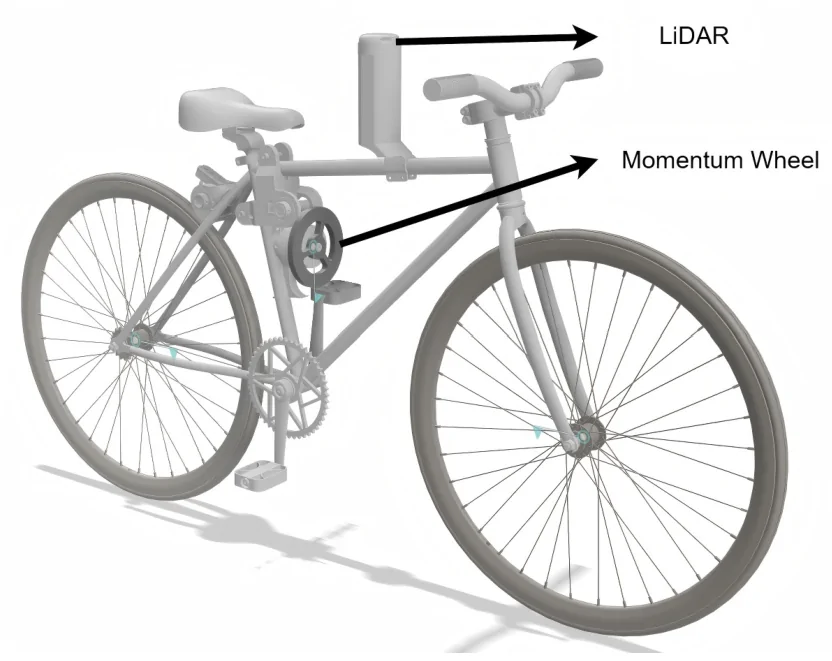
Autonomous bicycle modeling design diagram.

The autonomous driving research platform used in this paper is based on a standard bicycle with modifications, as shown in Figure 3.

To achieve active balance at low speed and while stationary, and to provide additional stabilizing torques in rugged terrain, the model integrates a self-built momentum wheel balancing system. The core components of this balancing system are mounted on the seat tube below the saddle, mainly comprising a high-speed DC brushless motor and a flywheel (momentum wheel) with large rotational inertia. By precisely controlling the motor’s acceleration and deceleration, the flywheel generates a reaction torque to actively regulate the vehicle’s roll attitude. Additionally, a LiDAR sensor is mounted horizontally at the front of the frame. Serving as the core environmental perception sensor, it collects range data from the horizontal plane ahead and outputs sparse distance vectors characterizing local obstacle distributions. Because the LiDAR focuses on planar geometric constraint detection, the real-time vehicle body pitch angle from an Inertial Measurement Unit (IMU) is additionally introduced to compensate for its limitations in three-dimensional terrain perception. This low-cost, high-versatility combination of “LiDAR for planar obstacle detection + IMU for dynamic terrain perception” effectively avoids complex three-dimensional point cloud processing while providing complete and necessary environmental state inputs for both the upper-layer RRT* planning algorithm and the PPO decision networks.

#### 3.2.1. Model Simplification and Coordinate Definition

The autonomous bicycle primarily consists of a frame, front and rear wheels, a steering mechanism, and a momentum wheel for active balance. To focus on the core control challenge of lateral balance, and considering that this paper primarily investigates stability at low speed and in rugged terrain, the following reasonable assumptions and simplifications are made:

1. Rigid body assumption: The frame, wheels, and momentum wheel are all assumed to be ideal rigid bodies, neglecting elastic deformation during motion.
2. Pure rolling assumption: Perfect contact between wheels and ground is assumed with no lateral slip (no-slip condition), satisfying nonholonomic constraints.
3. Inverted pendulum equivalence: In the lateral balance analysis, the frame and wheels are treated as a single rod with mass concentrated at the system’s combined center of mass; the momentum wheel is modeled as a rotating disk connected to the rod. The system’s motion in the lateral plane can be approximated as an Inverted Pendulum with a Reaction Wheel model.
4. Decoupling assumption: Although the bicycle’s forward motion and lateral motion are coupled, the longitudinal velocity is treated as a parameter when deriving the dynamic equations, with focus placed on the dynamic relationship between lateral tilt angle and momentum wheel speed.

**Figure 4:**
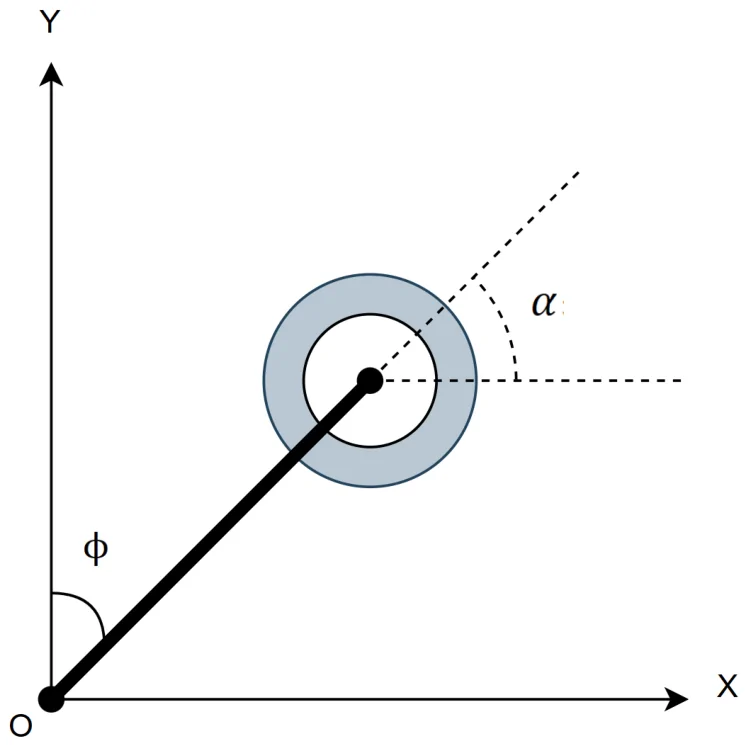
Simplified schematic of the autonomous bicycle model.

As shown in Figure 4, an inertial coordinate system and a body coordinate system are defined. The motion in the vertical plane is primarily governed by gravitational torque, centrifugal torque, and the reaction torque generated by the momentum wheel. A set of mutually independent generalized coordinates is chosen as $q={\left[\phi ,\;\alpha \right]}^{⊤}$, where the following apply.

ϕ: Roll angle of the vehicle body (pendulum rod) from the vertical direction, with the counterclockwise direction defined as positive. α: Spin angle of the momentum wheel relative to the vehicle frame. The relevant physical parameters and symbol definitions are listed in Table 1. Note that to accommodate the rugged terrain scenarios considered in this paper, the influence of terrain slope on the gravitational component is taken into account in the potential energy analysis.

#### 3.2.2. Energy Analysis and Lagrangian Function Construction

The Lagrangian function *L* is defined as the difference between the system’s total kinetic energy *T* and total potential energy *V*:

(1) L=T−V

System total kinetic energy *T*:

The system’s total kinetic energy consists of two parts: the kinetic energy of the vehicle body $T_{b}$ and the kinetic energy of the momentum wheel $T_{\omega }$. The vehicle body rotates about contact point *O* in the lateral plane:

(2) $$T_{b}=\frac{1}{2}I_{b}{\dot{\phi }}^{2}$$

The momentum wheel undergoes both a carry-along motion with the vehicle body and its own relative rotation. The absolute angular velocity of the momentum wheel in space is the vector sum of the vehicle body roll angular velocity $\dot{\phi }$ and the momentum wheel relative angular velocity $\dot{\alpha }$. Assuming the momentum wheel mounting axis is perpendicular to the vehicle body plane, the total kinetic energy can be expressed as the sum of translational and rotational kinetic energies:

(3) $$T_{\omega }=\frac{1}{2}m_{\omega }{\left(l_{\omega }\dot{\phi }\right)}^{2}+\frac{1}{2}I_{\omega }{\left(\dot{\phi }+\dot{\alpha }\right)}^{2}$$

The first term corresponds to the kinetic energy produced by the linear velocity of the momentum wheel’s center of mass swinging about the contact point *O*, and the second term corresponds to the kinetic energy of the momentum wheel rotating about its own axis. Letting the equivalent moment of inertia $J_{b}=I_{b}+m_{\omega }l_{\omega }^{2}$, the total kinetic energy is:

(4) $$T=T_{b}+T_{\omega }=\frac{1}{2}J_{b}{\dot{\phi }}^{2}+\frac{1}{2}I_{\omega }\left({\dot{\phi }}^{2}+2\dot{\phi }\dot{\alpha }+{\dot{\alpha }}^{2}\right)$$

System total potential energy *V*:

The system’s potential energy mainly comes from gravitational potential energy. Taking the horizontal plane at contact point *O* as the zero potential energy reference, and letting the equivalent gravity moment coefficient $M_{g}=m_{b}l_{b}+m_{\omega }l_{\omega }$:

(5) $$V=\left(m_{b}l_{b}+m_{\omega }l_{\omega }\right)\;g\cos\phi =M_{g}g\cos\phi$$

Note: In the rugged terrain scenarios (e.g., slope angle $\theta_{\mathrm{slope}}$) considered in this paper, the gravitational component acting on the restoring torque changes. Although the derivation here is based on a flat-ground assumption, in the subsequent simulation environment, terrain disturbances are introduced by modifying the vector direction of *g*.

Lagrangian function:

(6) $$L=T-V=\frac{1}{2}J_{b}{\dot{\phi }}^{2}+\frac{1}{2}I_{\omega }{\left(\dot{\phi }+\dot{\alpha }\right)}^{2}-M_{g}g\cos\phi$$

#### 3.2.3. Dynamic Equation Derivation

Applying the standard Lagrangian dynamic equation:

(7) $$\frac{d}{dt}\frac{\partial L}{\partial {\dot{q}}_{i}}-\frac{\partial L}{\partial q_{i}}=Q_{i}$$

where $Q_{i}$ is the generalized force. In this system, the generalized force for coordinate ϕ (vehicle body) is $Q_{\phi }=0$, since the motor torque is an internal force and no external lateral disturbance force is assumed. For coordinate α (momentum wheel), the motor torque drives the momentum wheel directly, so $Q_{\alpha }=M$.

Step 1: Equation for vehicle body roll angle ϕ:

(8) $$\left(J_{b}+I_{\omega }\right)\ddot{\phi }+I_{\omega }\ddot{\alpha }-M_{g}g\sin\phi =0$$

This equation reveals the coupling relationship between the vehicle body roll acceleration $\ddot{\phi }$ and the momentum wheel angular acceleration $\ddot{\alpha }$. Clearly, by changing $\ddot{\alpha }$ (i.e., controlling the acceleration and deceleration of the momentum wheel), a reaction torque can be generated to influence the change in $\ddot{\phi }$, thereby counteracting the gravitational torque $M_{g}g\sin\phi$.

Step 2: Equation for momentum wheel spin angle α:

(9) $$I_{\omega }\left(\ddot{\phi }+\ddot{\alpha }\right)=M$$

This equation shows that the motor output torque *M* directly determines the rate of change in the momentum wheel’s angular momentum.

#### 3.2.4. State-Space Modeling and Nonlinear Characteristics Analysis

Combining Equations (8) and (9) and eliminating $\ddot{\alpha }$, the explicit equation for vehicle body roll dynamics is:

(10) $$J_{b}\ddot{\phi }-M_{g}g\sin\phi =-M$$

This is a typical second-order nonlinear differential equation. Defining the system state vector $X={\left[\phi ,\;\dot{\phi },\;\alpha ,\;\dot{\alpha }\right]}^{⊤}$ and input vector u=[M], near the equilibrium point (ϕ≈0), the small-angle approximation sinϕ≈ϕ allows linearization.

The resulting linearized state-space equations $\dot{X}=AX+Bu$ are obtained by solving Equations (8) and (9) for $\ddot{\phi }$ and $\ddot{\alpha }$:

(11) $$A=\left[\begin{array}{cccc} 0 & 1 & 0 & 0\\ \frac{M_{g}g}{J_{b}} & 0 & 0 & 0\\ 0 & 0 & 0 & 1\\ -\frac{M_{g}g}{J_{b}} & 0 & 0 & 0 \end{array}\right],\;B=\left[\begin{array}{c} 0\\ -\frac{1}{J_{b}}\\ 0\\ \frac{1}{J_{b}}+\frac{1}{I_{\omega }} \end{array}\right]$$

However, the focus of this paper is on rugged terrain and narrow spaces where large tilt angles may occur beyond the linear regime, and terrain slope changes introduce time-varying external disturbance terms D(t). At that point, the simple linearized model $\dot{X}=AX+Bu$ can no longer accurately describe the dynamic behavior of the system, and the robustness of linear controllers will decrease significantly. Specifically, the gravitational term $M_{g}g\sin\phi$ in Equation (8) must be modified to complex three-dimensional projection components in the presence of slope $\theta_{\mathrm{slope}}$. When the vehicle executes large-curvature steering in narrow spaces, a significant centrifugal term $\frac{mv^{2}}{R}\cos\phi$ is also superimposed.

These highly nonlinear and coupled characteristics are the fundamental reason this paper abandons traditional PID or LQR control in favor of the dual-layer PPO deep reinforcement learning algorithm. The PPO algorithm can directly interact with the aforementioned nonlinear dynamics environment, mastering nonlinear control laws for maintaining balance in complex force fields through trial-and-error learning, thereby making up for the limitations of traditional linear modeling under extreme conditions.

It is worth clarifying the role of the simplified analytical model derived above within the overall framework. The model serves two specific purposes: first, it reveals the balance mechanism of the bicycle-momentum-wheel system—how the momentum-wheel torque generates the restoring moment that stabilizes the roll dynamics—which guides the choice of state variables, control inputs, and the structure of the reward functions for the lower-layer policy; second, it provides the linearized model on which the LQR baseline controller used for comparison is designed. The dual-layer PPO policy itself does not rely on this simplified model: all training is conducted in PyBullet, where friction coefficients, wheel-ground contact conditions, and terrain parameters are set explicitly and the simulated system is treated as a black box. Consequently, conclusions drawn from the simplified analysis are used only to motivate the control design, while the effectiveness of the proposed controller is established by the simulation experiments in Section 4.

### 3.3. Global Path Planning

In unstructured narrow terrain, traditional gradient-based planning algorithms are highly prone to local minima. Although the standard RRT algorithm has probabilistic completeness for finding feasible paths in large spaces, the geometric paths it generates are typically composed of discrete line segments without asymptotic optimality, resulting in lengthy and tortuous routes. Furthermore, the nonholonomic constraints of autonomous bicycles prohibit instantaneous lateral translation and require satisfaction of curvature continuity conditions. Therefore, this paper proposes a two-stage global planning strategy combining the RRT* algorithm with cubic B-splines. The main reason for choosing this combination lies in the complementary strengths and weaknesses of the two components. On the one hand, RRT* retains the probabilistic completeness of RRT in large, cluttered spaces and further introduces asymptotic optimality through parent reselection and rewiring, which progressively shortens the path and reduces the number of turns; its output, however, remains piecewise linear with discontinuous curvature at the nodes. Such paths cannot be tracked directly by a bicycle, whose nonholonomic constraints forbid sharp cornering—direct tracking would force frequent stop-and-turn maneuvers or even cause a loss of balance. On the other hand, cubic B-spline smoothing converts the RRT* path into a $C^{2}$ continuity reference trajectory, so that both the path and its curvature vary smoothly and can be tracked stably by the downstream dual-layer PPO controller. In short, RRT* guarantees a globally feasible and near-optimal corridor, while the cubic B-spline guarantees that the reference trajectory is dynamically trackable for the bicycle. First, the RRT* algorithm searches for a collision-free piecewise-linear path with asymptotic optimality in the configuration space; then cubic B-spline smoothing converts the discrete waypoints into a reference trajectory satisfying $C^{2}$ continuity, thus meeting both obstacle avoidance requirements and vehicle dynamics characteristics.

#### 3.3.1. Problem Formulation and Configuration Space Definition

The path planning problem is defined as a geometric search and cost optimization problem in two-dimensional configuration space ($C\in R^{2}$). Let $C_{\mathrm{free}}$ denote the collision-free free space and $C_{\mathrm{obs}}$ denote the obstacle region. The vehicle’s center-of-mass state in the global planning stage is simplified to two-dimensional coordinates q=(x,y). Because the bicycle model cannot execute paths with sharp corners in actual driving, the global planner’s objective is to find a feasible initial path $P=\{q_{1},q_{2},\ldots ,q_{n}\}$ from start $q_{\mathrm{start}}$ to goal $q_{\mathrm{goal}}$ in $C_{\mathrm{free}}$ by minimizing the Euclidean distance cost, which is defined as the cumulative path length $J\left(P\right)=\sum_{i=1}^{n-1}{∥q_{i+1}-q_{i}∥}_{2}$, i.e., the sum of the Euclidean distances between every pair of consecutive nodes $q_{i}$ and $q_{i+1}$ along the path from $q_{\mathrm{start}}$ to $q_{\mathrm{goal}}$. Minimizing this cost means that RRT* asymptotically converges to the shortest collision-free path in terms of total travel distance, providing a high-quality reference baseline for subsequent curve smoothing.

It should be emphasized that the two-dimensional formulation is a deliberate division of labor rather than an omission of the three-dimensional terrain. In our application scenario, the global planning layer works on a known two-dimensional map; the task of this layer is to guarantee a geometrically feasible corridor that reaches the goal without colliding with these structural elements, while unmapped local obstacles are perceived online by the single-line LiDAR and avoided by the local control layer (Section 3.4). The three-dimensional aspects of rugged terrain—elevation changes, slope traversability, and pitch constraints—are deliberately handled at the local control layer: the upper-layer PPO network takes the vehicle body pitch angle as part of its observation and is trained extensively on stairs, slopes, and uneven surfaces, so that slope traversal and pitch-constraint satisfaction are enforced by the learned controller during execution (Section 3.4). This division of labor keeps the global planning problem tractable while preserving the vehicle’s ability to traverse rugged terrain.

#### 3.3.2. RRT* Tree Expansion and Asymptotically Optimal Rewiring

To address the suboptimal path issue generated by the standard RRT algorithm, this algorithm incorporates the core mechanism of RRT* [46], which involves parent node reselection and random tree reconfiguration. The core workflow of a single iteration of the algorithm is as follows:

Target-biased Sampling: When randomly sampling state $q_{\mathrm{rand}}$ in the C-space, the goal point $q_{\mathrm{goal}}$ is sampled with probability 10%, and uniform global sampling is applied otherwise. This accelerates algorithm convergence.

Fixed-step Steering (Steer): The nearest node $q_{\mathrm{nearest}}$ in the tree is found, and a new node $q_{\mathrm{new}}$ is generated by extending from $q_{\mathrm{nearest}}$ toward $q_{\mathrm{rand}}$ by a fixed step length.

Optimal Parent Selection (Choose Parent): If $q_{\mathrm{new}}$ passes collision detection, the algorithm defines a dynamic search radius *r* around $q_{\mathrm{new}}$:

(12) $$r=\gamma \sqrt{\frac{\log(N)}{N}}$$

where *N* is the current number of nodes in the tree and γ is a tuning coefficient (set to 50.0 in this paper). The algorithm selects the collision-free node in the neighbor set $Q_{\mathrm{near}}$ that minimizes the cumulative cost to $q_{\mathrm{new}}$ as the final parent node.

Tree Rewiring: After adding $q_{\mathrm{new}}$ to the tree, the algorithm traverses $Q_{\mathrm{near}}$ again. If the cost of reaching any neighbor $q_{\mathrm{near}}$ via $q_{\mathrm{new}}$ is less than its current cost and the path is collision-free, the neighbor’s parent is reassigned to $q_{\mathrm{new}}$. This local topological adjustment enables the tree to continuously approach the global optimum during iteration.

The complete RRT* planning workflow, from tree initialization to B-spline smoothing, is summarized in Algorithm 1.
**Algorithm 1** RRT*-based global path planning with cubic B-spline smoothing.**Require:** 
Start point $q_{\mathrm{start}}$, goal point $q_{\mathrm{goal}}$, configuration space C with obstacle region $C_{\mathrm{obs}}$, step size η, goal-bias probability $p_{g}=0.1$, maximum iteration number $N_{\max}$, goal tolerance ε**Ensure:** 
$C^{2}$-continuous reference trajectory C(u)   1:$T\leftarrow \{q_{\mathrm{start}}\}\;$▹ Initialize the search tree   2:**for** i=1 **to** $N_{\max}$ **do**   3:    $q_{\mathrm{rand}}\leftarrow \mathrm{Sample}\left(C,\;p_{g},\;q_{\mathrm{goal}}\right)\;$▹ Goal-biased sampling   4:    $q_{\mathrm{nearest}}\leftarrow \mathrm{NearestNeighbor}\left(T,\;q_{\mathrm{rand}}\right)$   5:    $q_{\mathrm{new}}\leftarrow \mathrm{Steer}\left(q_{\mathrm{nearest}},\;q_{\mathrm{rand}},\;\eta \right)\;$▹ Fixed-step extension   6:    **if** $\mathrm{CollisionFree}(q_{\mathrm{nearest}},\;q_{\mathrm{new}})$ **then**   7:        $r\leftarrow \gamma \sqrt{\log N/N}$; $Q_{\mathrm{near}}\leftarrow \mathrm{Near}\left(T,\;q_{\mathrm{new}},\;r\right)\;$▹ Dynamic search radius   8:        $q_{\mathrm{parent}}\leftarrow \mathrm{ChooseParent}\left(Q_{\mathrm{near}},\;q_{\mathrm{new}}\right)\;$▹ Minimum-cost parent selection   9:        $T\leftarrow T\cup \{\left(q_{\mathrm{parent}},\;q_{\mathrm{new}}\right)\}\;$▹ Add node and edge 10:        $\mathrm{Rewire}(T,\;Q_{\mathrm{near}},\;q_{\mathrm{new}})\;$▹ Asymptotic optimization 11:        **if** $∥q_{\mathrm{new}}-q_{\mathrm{goal}}∥\leq \varepsilon$ **and** $\mathrm{CollisionFree}(q_{\mathrm{new}},\;q_{\mathrm{goal}})$ **then** 12:           Connect $q_{\mathrm{goal}}$ to T; **break** 13:        **end if** 14:    **end if** 15:**end for** 16:$P_{\mathrm{raw}}\leftarrow \mathrm{Backtrack}\left(T,\;q_{\mathrm{goal}}\right)\;$▹ Raw waypoint sequence 17:$C\left(u\right)\leftarrow \mathrm{CubicBSpline}\left(P_{\mathrm{raw}}\right)\;$▹$C^{2}$-continuous smoothing, Equation (13) 18:**return** C(u)

#### 3.3.3. Path Smoothing via Cubic B-Splines

Although the RRT*-generated path satisfies kinematic constraints, the stochastic sampling nature results in jitter and unnecessary convolutions. To provide high-quality reference signals for the upper-layer control strategy, cubic B-spline smoothing is applied to the raw waypoints $P_{\mathrm{raw}}=\left\{q_{0},q_{1},\ldots ,q_{N}\right\}$. The B-spline curve C(u) is defined as a linear combination of n+1 control points $P_{i}$:

(13) $$C\left(u\right)=\sum_{i=0}^{n}N_{i,k}\left(u\right)\;P_{i},\;u\in \left[u_{k},\;u_{n+1}\right]$$

where $N_{i,k}\left(u\right)$ are the *k*-th order basis functions computed by the Cox-de Boor recursion formula. Cubic B-splines possess $C^{2}$ continuity, ensuring smooth transitions of position, velocity, and acceleration (curvature).

**Figure 5:**
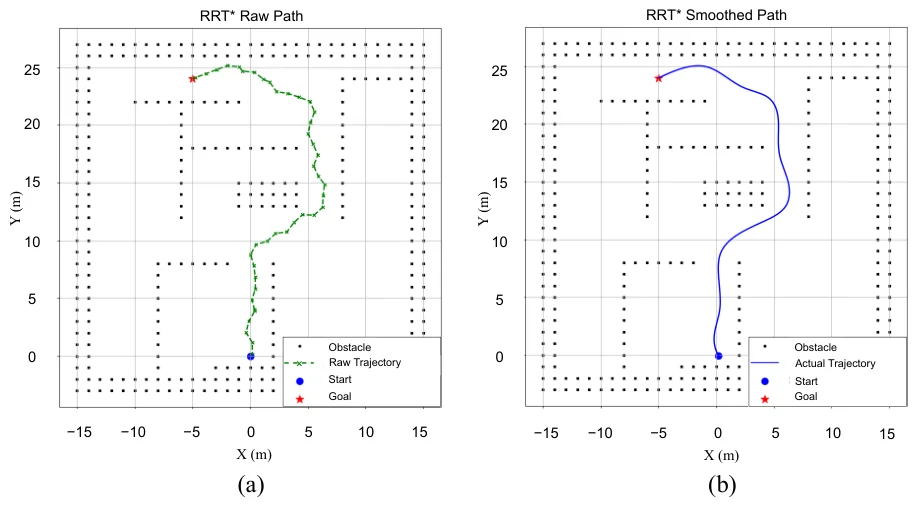
Comparison of path planning trajectories in an obstacle-cluttered environment. (**a**) The raw path generated by the traditional RRT* algorithm. (**b**) The smoothed path optimized by the B-spline curve.

Figure 5 shows the trajectory comparison before and after smoothing. The smoothed path retains sufficient safety margin at narrow bends while removing high-frequency noise from the original path, significantly reducing the tracking error of the lower-level controller.

It should be noted that geometric smoothness alone does not guarantee full dynamic feasibility; in this work, feasibility is ensured at two levels. At the planning level, RRT* plans a collision-free geometric path in the free space, and the cubic B-spline smoothing converts it into a $C^{2}$-continuous reference trajectory. At the execution level, the remaining dynamic requirements—steering-angle and steering-rate limits, roll stability, and terrain-dependent speed adjustment—are enforced by the dual-layer PPO controller: the lower layer maintains roll stability via the momentum wheel, the policy moderates speed and steering in response to terrain and curvature, and the reward design penalizes proximity to obstacles so that a safe clearance is maintained during tracking. Therefore, the global layer provides geometric feasibility, while dynamic and nonholonomic feasibility is realized by the learned local controller rather than by the smoothed path alone.

### 3.4. Dual-Layer PPO Controller Design

Given the high-dimensional continuous state space and complex nonlinear dynamics of the autonomous bicycle system, traditional end-to-end control methods are difficult to simultaneously handle the flexibility of local path planning and the robustness of anti-disturbance balance.The traditional methods referred to here fall into two categories. The first category is end-to-end learning-based navigation controllers (e.g., DDPG- or SAC-style policies that map raw observations directly to control commands). Without access to global map information, such policies struggle with long-distance navigation in cluttered, constrained environments and are prone to local minima and deadlock regions such as U-shaped traps. The second category is classical linear model-based controllers (e.g., PID and LQR), which rely on dynamic models linearized near the equilibrium point; their performance degrades significantly under the large tilt angles, time-varying terrain disturbances, and strongly coupled nonlinear dynamics encountered on rugged terrain. Quantitative comparisons against representative baselines of both categories (RRT* + LQR, end-to-end single-layer PPO, and RRT* + single-layer PPO) are provided in Section 4. This paper proposes a dual-layer hierarchical control architecture based on Proximal Policy Optimization (PPO), decomposing the complex control problem into two independent Markov Decision Processes (MDPs) corresponding to upper-level kinematic decision-making and lower-level dynamic execution.

#### 3.4.1. Hierarchical Cooperation Logic of the Control Architecture

**Figure 6:**
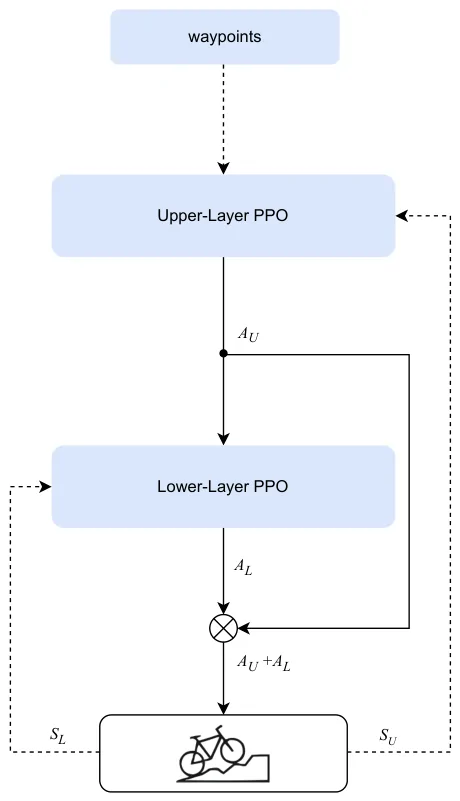
Dual-layer PPO network framework diagram.

As illustrated in Figure 6, the control task is divided into two hierarchical levels.

Upper-Layer PPO. Its core task is to formulate macroscopic motion strategies based on three-dimensional environmental features. It does not directly handle low-level balance, but instead guides the lower-layer controller through desired compensation quantities $A_{U}=\left[\beta_{U},v_{U}\right]$, where $\beta_{U}$ is the desired steering angle and $v_{U}$ is the desired travel velocity, to complete obstacle avoidance and terrain traversal actions.

Lower-Layer PPO. Its core task is to maintain vehicle balance and respond to basic motion commands. It focuses on high-frequency attitude adjustment, uses the precession or reaction torque produced by the momentum wheel to assist balance, and superimposes the kinematic desired compensation quantities from the upper layer to produce the final action $A_{L}+A_{U}$, where $A_{L}=\left[\beta_{L},v_{L},T_{L}\right]$ contains handlebar angle control $\beta_{L}$, vehicle speed control $v_{L}$, and momentum wheel torque control $T_{L}$. The goal is to execute the upper-layer terrain traversal commands as precisely as possible while maintaining dynamic balance.

It should be clarified that the two layers are not completely decoupled: the final steering and velocity commands are the superposition of the two layers’ outputs, and the waypoint-error terms are included in both state vectors. Command conflicts are mitigated by the training scheme and the action-space design rather than by structural isolation. First, the lower-layer policy is pre-trained across a wide range of speeds and steering commands before upper-layer training starts (Section 3.4.4), so it learns to maintain balance under diverse command interventions; the upper layer is then trained on top of the converged lower-layer executor and learns to issue commands that the lower layer can realize without destabilizing the vehicle. Second, the action outputs of both networks are bounded: the normalized actions are mapped to the physical limits of the steering mechanism, the drive motor, and the momentum wheel, so the superimposed commands always remain within the actuators’ feasible range and action saturation conflicts are avoided. Third, the waypoint-error terms shared by both state vectors provide a common reference that keeps the two layers’ objectives aligned. Both layers operate at the same control frequency of 25 Hz, synchronized with the 0.04 s simulation time step (Table 2): the upper layer updates its desired commands once per simulation step, while the lower layer reacts to fast attitude dynamics at every control step.

#### 3.4.2. Upper-Layer Controller

The upper-layer controller interacts directly with the environment, focusing on the geometric feasibility of the local space and terrain adaptability. It does not directly handle the inverted pendulum balance of the vehicle body, but instead determines ideal kinematic commands that satisfy obstacle avoidance and path tracking constraints based on environmental perception information.

State Space ($S_{U}$): The upper-layer observation vector $o_{U}\in R^{64}$ aims to comprehensively perceive local environmental geometric features:

(14) $$o_{U}=\left[L_{\mathrm{scan}},\;d_{\mathrm{waypoint}},\;\psi_{\mathrm{waypoint}},\;\delta ,\;\phi_{p}\right]$$

where $L_{\mathrm{scan}}\in R^{60}$ is a normalized 60-dimensional sparse LiDAR distance vector characterizing the obstacle distribution in the forward sector; $d_{\mathrm{waypoint}}$ and $\psi_{\mathrm{waypoint}}$ are the Euclidean distance and heading angle deviation to the nearest waypoint; δ is the current steering angle, ensuring decision continuity; and $\phi_{p}$ is the current vehicle body pitch angle, reflecting real-time terrain gradient changes and road surface irregularities. To support the local tracking capability of the lower-layer PPO, the upper-layer network also passes $d_{\mathrm{waypoint}}$ and $\psi_{\mathrm{waypoint}}$ as shared signals to the lower layer. This sharing mechanism provides the lower layer with a directional reference and prevents it from deviating from the correct heading due to excessive focus on balance.

Action Space ($A_{U}$):

(15) $$A_{U}=\left[\beta_{U},\;v_{U}\right]$$

where $\beta_{U}$ is the desired steering angle, reflecting the steering posture the system should maintain for path tracking and obstacle avoidance at the current moment, and $v_{U}$ is the desired travel velocity. This is the key decision variable for the upper layer when dealing with rugged terrain: when a steep slope or continuous stairs is detected, the upper layer actively outputs a lower desired speed to reduce impact loads; in flat and open areas, it outputs a higher speed to improve passage efficiency. This pair $\left(\beta_{U},v_{U}\right)$ is passed to the lower layer as a kinematic reference and is not used directly to drive the motors.

Reward Function ($R_{U}$):

(16) $$R_{U}=R_{\mathrm{waypoint}}+R_{\mathrm{collision}}+R_{\mathrm{approach}}+R_{\mathrm{pitch}}+R_{\mathrm{time}}$$

Sparse goal reward ($R_{\mathrm{waypoint}}$): Upon successfully reaching the target (error <0.5 m), a high reward of +20,000 is given to reinforce the task completion motivation. Safety penalty ($R_{\mathrm{collision}}$): If a collision occurs (LiDAR minimum value below threshold or physical contact), a penalty of −10,000 is given and the episode terminates. Dense approach reward ($R_{\mathrm{approach}}$):

(17) $$R_{\mathrm{approach}}=-k_{d}\cdot d_{\mathrm{waypoint}}-k_{\psi }\cdot \left|\psi_{\mathrm{waypoint}}\right|$$

with coefficients $k_{d}=0.05$, $k_{\psi }=0.1$, encouraging the vehicle to continuously approach the target. Terrain stability penalty ($R_{\mathrm{pitch}}$):

(18) $$R_{\mathrm{pitch}}=\left\{\begin{array}{cc} -k_{p}\cdot \left|\phi_{p}\right|, & \mathrm{if}\;\left|\phi_{p}\right|>P_{\mathrm{threshold}}\\ 0, & \mathrm{otherwise} \end{array}\right.$$

with $k_{p}=10$. By penalizing pitch angles $|\phi_{p}|$ that exceed the safe threshold $P_{\mathrm{threshold}}$, the controller is forced to maintain a relatively stable vehicle posture by adjusting the steering path or reducing the instantaneous speed when severe terrain undulation is detected. Time-step penalty ($R_{\mathrm{time}}$): −1 per step, encouraging shorter paths.

#### 3.4.3. Lower-Layer Controller

The lower-layer controller addresses the high-dimensional nonlinear dynamic problem of the bicycle. It operates as a pre-trained “expert model” that maintains vehicle balance and controls speed toward target points.

State Space ($S_{L}$): The lower-layer observation vector $o_{L}\in R^{8}$ focuses on the vehicle body’s own dynamic state:

(19) $$o_{L}=\left[\phi ,\;\dot{\phi },\;\delta ,\;\dot{\delta },\;v,\;\omega_{\mathrm{fw}},\;d_{\mathrm{waypoint}},\;\psi_{\mathrm{waypoint}}\right]$$

containing key balance states: body roll angle ϕ, roll angular velocity $\dot{\phi }$, handlebar steering angle δ, handlebar steering angular velocity $\dot{\delta }$, vehicle speed *v*, and momentum wheel speed $\omega_{\mathrm{fw}}$. The variables $d_{\mathrm{waypoint}}$ and $\psi_{\mathrm{waypoint}}$ serve only as directional guidance for the lower-layer balance control, preventing it from deviating from the correct heading in pursuit of vehicle stability.

Action Space ($A_{L}$):

(20) $$A_{L}=\left[\beta_{L},\;v_{L},\;T_{L}\right]$$

corresponding to handlebar angle control $\beta_{L}$, vehicle speed control $v_{L}$, and momentum wheel torque control $T_{L}$, where $T_{L}$ denotes the normalized momentum-wheel torque command output by the lower-layer policy, which is mapped to the physical torque range of the momentum wheel during execution.

Reward Function ($R_{L}$): The lower-layer reward consists of a balance term $R_{\mathrm{balance}}$ and a goal-reaching term $R_{\mathrm{goal}}$:

(21) $$R_{L}=R_{\mathrm{balance}}+R_{\mathrm{goal}}$$

(22) $$R_{\mathrm{balance}}=\left\{\begin{array}{cc} -10.0, & \mathrm{if}\;|\phi |>0.2\;\mathrm{rad}\;\left(\mathrm{fallen}\right)\\ -|\phi |, & \mathrm{otherwise} \end{array}\right.$$

where −|ϕ| aims to minimize the roll angle; when |ϕ|>0.2 rad the vehicle is considered to have fallen, incurring a penalty of −10.0. The goal-reaching term $R_{\mathrm{goal}}=+100$ is given once when the vehicle reaches the commanded target point.

It is worth clarifying how the lower-layer policy learns appropriate speed and steering behaviors under a reward dominated by the roll-angle objective. The lower-layer policy is trained as a balance expert in target-reaching episodes: its observation contains the waypoint errors $d_{\mathrm{waypoint}}$ and $\psi_{\mathrm{waypoint}}$, and the training task requires the vehicle to approach the waypoint while remaining balanced. Within this task design, the roll-angle objective in Equation (21) dominates the balance behavior, while the speed and steering behaviors are shaped jointly by the waypoint-guidance signals, the goal-reaching reward $R_{\mathrm{goal}}$, and the stability requirement—any aggressive speed or steering command that would destabilize the vehicle is suppressed by the roll penalty, so the policy learns coordinated speed and steering behaviors together with attitude stabilization.

#### 3.4.4. Network Architecture and Training

Hierarchical Training Strategy. Due to the hierarchical dependency between the two-layer networks, a “bottom-up” staged curriculum training strategy is adopted:

Stage 1: Train the lower-layer PPO network in an obstacle-free flat environment, enabling it to maintain dynamic vehicle stability using the steering mechanism and momentum wheel. Stage 2: Freeze the lower-layer network parameters once it converges and can stably control bicycle balance, embedding it as a stable low-level actuator in the environment. Stage 3: Train the upper-layer PPO network. At this stage, the upper-layer network focuses on processing LiDAR information and global path planning, learning to generate guidance commands that can be effectively executed by the lower-layer network. This training order matches the actual operational logic of the system, ensuring that lower-level control robustness is the prerequisite for the effectiveness of upper-level decision planning, and effectively avoids policy oscillation caused by lower-level instability during the early stages of joint training.

**Figure 7:**
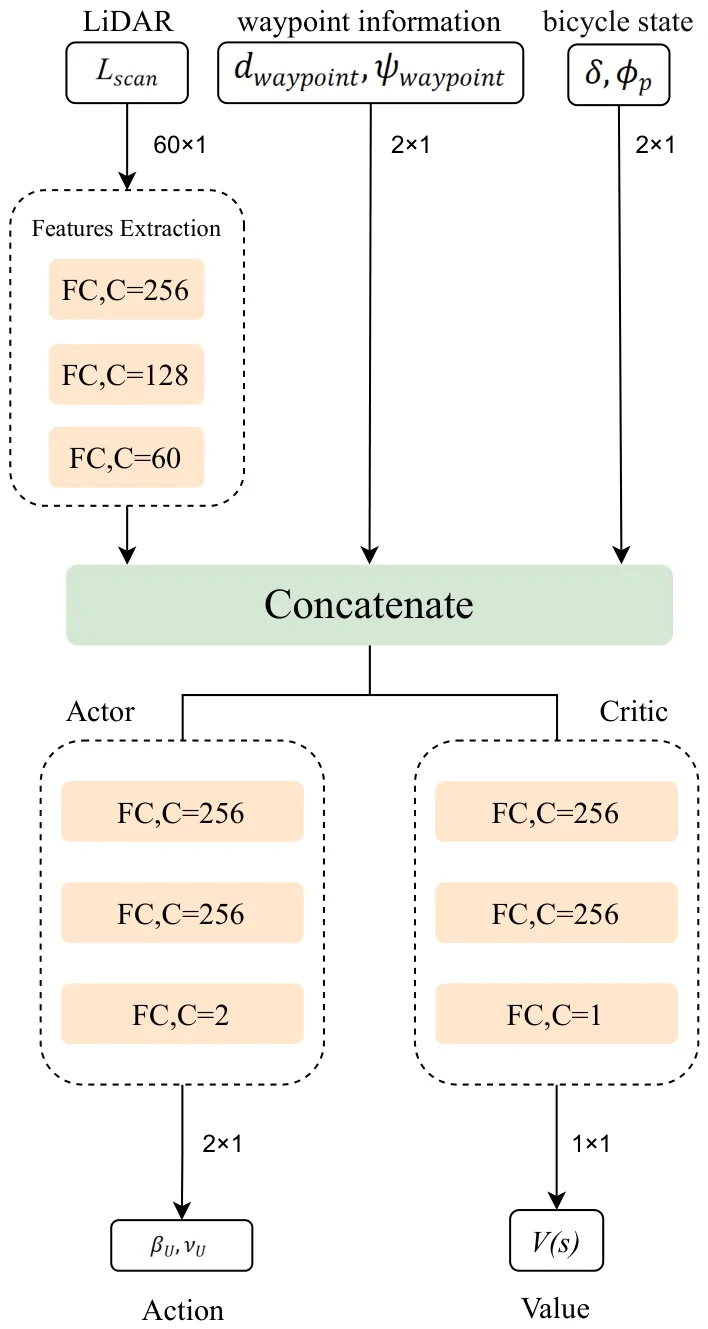
Upper-level network architecture diagram.

Upper-Level Network Architecture. As shown in Figure 7, the upper-level network adopts the standard Actor–Critic architecture. For the high-dimensional perception information, a feature extraction module using two fully connected layers with ReLU activations is designed for the 60-dimensional sparse LiDAR vector. The extracted environmental geometric feature vector and navigation state vector are fused in a concatenation layer to form a 64-dimensional comprehensive feature vector. The Actor network contains three fully connected hidden layers with [256, 256, 2] neurons; ReLU activations enhance the network’s ability to represent nonlinear obstacle boundaries. The output layer has 2 neurons mapped through a Tanh function to the [−1,1] interval, representing the normalized desired steering angle and desired speed. The Critic network has similar topology but outputs a scalar V(s) for advantage function computation.

**Figure 8:**
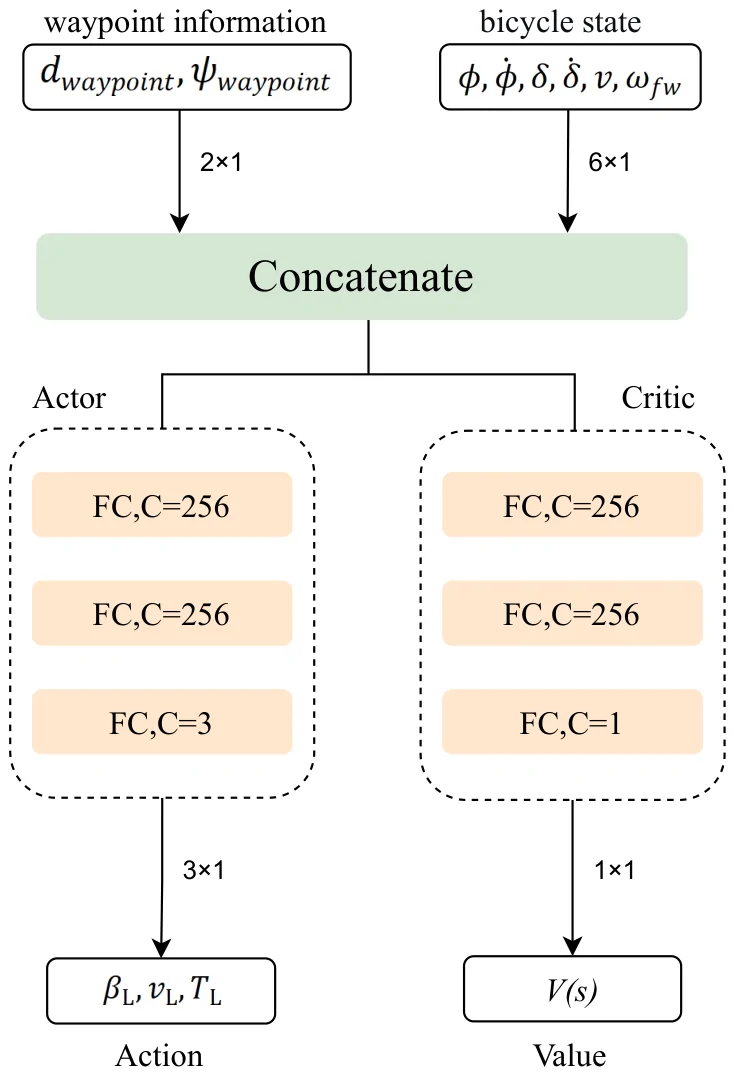
Lower-level network architecture diagram.

Lower-Level Network Architecture. As shown in Figure 8, the lower-level network design focuses on high-frequency dynamic response. Because the input feature dimension is low and the physical meaning is clear, the lower-layer network feeds the 8-dimensional raw state vector directly into a Multi-Layer Perceptron (MLP). Both Actor and Critic networks share the [256, 256] hidden layer configuration. The main difference is that the Actor network’s output layer has 3 neurons, corresponding to handlebar angle control $\beta_{L}$, vehicle speed control $v_{L}$, and momentum wheel torque control $T_{L}$.

## 4. Experimental Verification and Performance Analysis

### 4.1. Experimental Setup

#### 4.1.1. Simulation Platform Construction and Dynamic Modeling

All experimental evaluations in this study were conducted using the high-fidelity physics simulator PyBullet (version 3.2.7). As a widely adopted open-source simulator in robotics and reinforcement learning, PyBullet provides a reliable virtual environment for controlling an autonomous bicycle, which is a typical underactuated and strongly nonlinear system, through its efficient rigid-body dynamics solver, precise collision detection, and detailed frictional contact modeling. Compared with other simulation tools, PyBullet natively supports a Python (version 3.11) interface and integrates seamlessly with mainstream deep learning frameworks such as PyTorch (version 2.6.0) and reinforcement learning libraries, greatly reducing the technical barriers to algorithm development and iteration.

**Figure 9:**
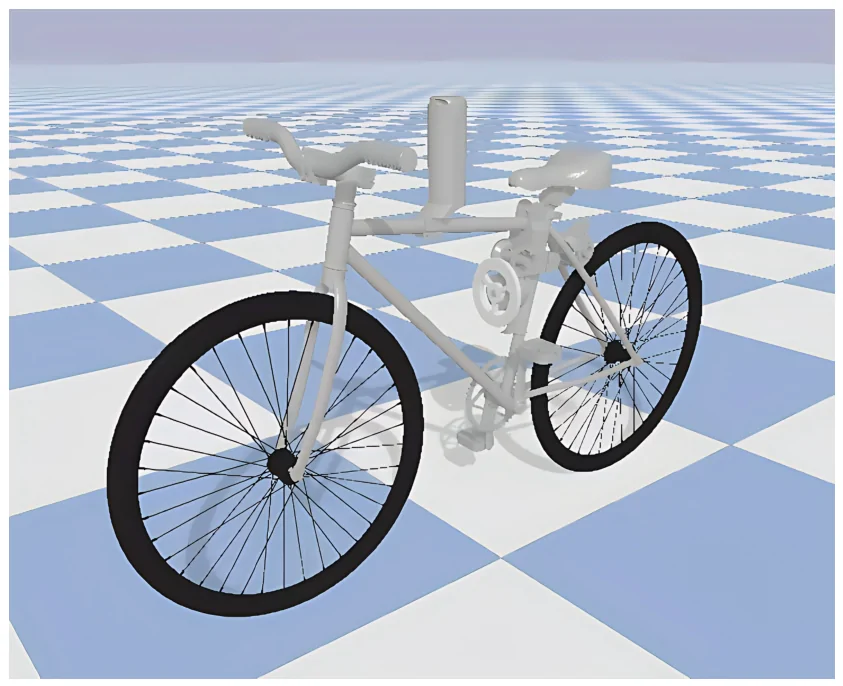
Bicycle model in PyBullet.

To address the inherent low-speed instability of the bicycle and endow the agent with autonomous navigation capabilities, specific hardware modifications are made to the model, as shown in Figure 9. First, a high-speed momentum wheel (mass: 0.5 kg, maximum no-load speed: 3000 RPM) is rigidly mounted below the saddle; serving as the core actuator for self-balancing, it generates reaction torques via angular momentum variations to actively counteract roll disturbances. Second, a 30 Hz single-line LiDAR sensor is deployed at the frame’s center of mass to output real-time polar coordinate distance sequences, providing the spatial geometric information required for obstacle avoidance decision-making. Additionally, the standard rear-wheel drive motor (maximum speed: 2000 RPM) and front fork steering joint are retained to execute longitudinal speed tracking and heading adjustments, respectively. The simulation environment’s physical parameters are calibrated according to the physical prototype (see Table 2). The ground friction coefficient is set to 0.6 to simulate dry asphalt road surfaces, and the gravitational acceleration is set to −9.8 m/s^2^. To maximize training efficiency, the DIRECT mode of PyBullet is used throughout, disabling the graphics rendering pipeline and significantly reducing computational overhead.

#### 4.1.2. Reinforcement Learning Training Framework and Procedure

This study uses Stable-Baselines3 (version 2.6.0) (SB3) as the core reinforcement learning training framework, built on PyTorch and providing efficient implementations of multiple mainstream algorithms including PPO. A custom Gym (version 0.23.1)-compatible environment class BicycleEnv is defined with standard reset, step, state acquisition, and action execution interfaces, ensuring seamless integration of PyBullet physics simulation into SB3’s training loop. PPO is selected as the base optimizer due to its clipped surrogate objective mechanism, which effectively prevents performance collapse during training and is particularly well-suited for continuous control tasks requiring smooth and stable actions. All training tasks are completed on a local workstation with Intel Core i5-11400 CPU, 16 GB RAM, and NVIDIA GeForce RTX 1650 (4 GB VRAM).

### 4.2. Rugged Terrain Adaptability Experiment

#### 4.2.1. Rugged Terrain Scenario Construction

This experiment is designed to evaluate the performance gain of the hierarchical control architecture compared to the non-hierarchical baseline when handling strongly nonlinear dynamic disturbances, with a primary focus on assessing the robustness of the low-level control strategy. To eliminate the interference of the global path planning module on the experimental conclusions, neither of the two comparative algorithms incorporates the RRT* planner, aiming to purely evaluate the inherent differences between the control architectures. The comparative algorithms are configured as follows:

Single-layer PPO (Baseline): An end-to-end control architecture employing a single PPO network. This network receives the complete state observation of the vehicle and simultaneously outputs three-dimensional control commands: steering angle, vehicle speed, and momentum wheel speed. A single policy implicitly handles both navigation decision-making and dynamic balance tasks, which introduces an inherent multi-task conflict.

Dual-layer PPO (Proposed): The dual-layer hierarchical PPO architecture proposed in this paper. The upper-layer network focuses on speed and heading planning at the kinematic level, while the lower-layer network, based on real-time IMU feedback, coordinates the steering mechanism and the momentum wheel, exclusively dedicated to maintaining the dynamic balance of the vehicle body. The two networks interact in a hierarchically coupled manner via desired kinematic commands.

**Figure 10:**
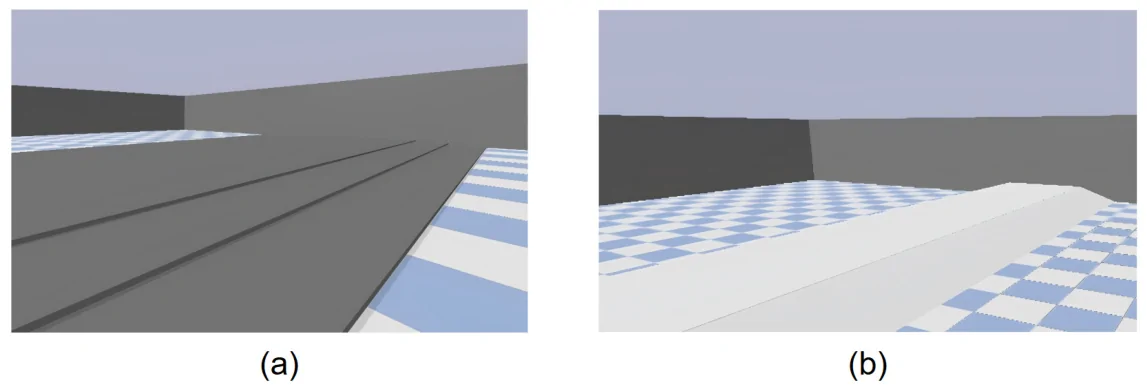
Staircase terrain (**a**) and slope terrain (**b**).

The evaluation metrics include Task Success Rate and Average Completion Time. To simulate the real dynamic challenges faced by autonomous bicycles navigating on unstructured surfaces, two types of typical rugged terrain test scenarios are constructed in the PyBullet simulation environment, as illustrated in Figure 10.

Continuous step scenario: A continuous step scenario with step heights h=5 cm and h=10 cm arranged along the direction of movement. When the wheel impacts a stair edge, the vehicle body experiences instantaneous impact loads causing violent fluctuations in roll and pitch angles. This scenario primarily evaluates the controller’s ability to suppress high-frequency impact disturbances from discontinuous ground contact.

Steep slope scenarios: Create steep slope scenarios with inclination angles of $10^{\circ }$, $15^{\circ }$, and $20^{\circ }$. The bicycle must continuously overcome the gravitational component along the slope surface while precisely coordinating rear-wheel drive torque and momentum wheel reaction torque under limited friction constraints, to prevent the front wheel from lifting due to excessive drive force or the vehicle from tipping over due to accumulated lateral gravitational components. This scenario primarily evaluates the controller’s tracking and compensation capability for persistent low-frequency external disturbances.

The test scenarios are configured with a fixed start point and random goal points (with the target region defined as x∈[−15,15], y=23), and each scenario is independently executed for 200 trials. Task success is defined as the system successfully completing the point-to-point traversal within the prescribed time limit, free of collisions and boundary violations, while simultaneously maintaining low-level dynamic balance.

#### 4.2.2. Rugged Terrain Experimental Results and Analysis

Table 3 summarizes the test results of single-layer PPO and dual-layer PPO under the five terrain parameter configurations described above.

Overall, the dual-layer PPO significantly outperforms the single-layer PPO baseline in all test scenarios, with the performance gap continuously widening as terrain difficulty increases.

In the continuous stairs scenario, the success rates of the single-layer PPO under h=5 cm and h=10 cm are merely 30.4% and 17.6%, respectively, indicating its extremely limited capability to suppress impact disturbances. Under the same conditions, the dual-layer PPO achieves 99.4% and 97.7%, respectively, demonstrating strong robustness against high-frequency impact disturbances.

In the steep slope scenario, the success rate of the single-layer PPO drops sharply from 59.5% to 16.2% as the slope angle increases, showing a precipitous decline. This reveals a significant robustness bottleneck in the single-layer architecture under conditions of continuous disturbance accumulation. The dual-layer PPO maintains success rates of 94.8%, 85.5%, and 69.4% on $10^{\circ }$, $15^{\circ }$, and $20^{\circ }$ slopes, respectively; although the rate decreases with escalating difficulty, the overall performance remains substantially higher than the baseline level.

The average completion time of the dual-layer PPO is approximately 1.0–2.4 s longer than that of the single-layer PPO. This is because the lower-layer controller imposes active constraints on the vehicle speed during attitude adjustment, making a moderate sacrifice in traversal efficiency in exchange for dynamic stability, a tradeoff with explicit significance for safety engineering.

The fundamental reason for the performance discrepancy lies in the fact that the single-layer PPO undertakes two types of tasks—navigation decision-making and dynamic balancing—that conflict in terms of time scales and optimization objectives. When subjected to stair impacts, the single network must simultaneously respond to attitude instability signals and path tracking errors within an extremely short time window. The superposition of these two types of gradient information leads to oscillatory action outputs, and even erroneous decisions where maintaining heading is prioritized, thereby delaying balance correction. The dual-layer architecture eliminates this conflict through explicit hierarchical decoupling: the lower-layer controller independently responds to high-frequency attitude disturbances and can rapidly mobilize the momentum wheel to generate reverse compensation torque without interference from upper-layer navigation commands; concurrently, the upper-layer controller focuses on kinematic planning at a lower decision frequency, outputting smooth desired commands to prevent aggressive maneuvers from interfering with low-level stability.

Furthermore, an analysis of the action sequences reveals that in the $20^{\circ }$ slope scenario, the single-layer PPO tends to compensate for the lateral gravitational component by substantially turning the handlebars. This not only increases rolling resistance but also further disrupts longitudinal stability. In contrast, the dual-layer PPO prioritizes utilizing the dynamic adjustment of the momentum wheel’s angular velocity to counteract the roll torque, making only minor corrections to the handlebar angle when necessary. This approach more closely aligns with the physical characteristics of bicycle lateral dynamics and preserves a larger stability margin under extreme conditions.

It is worth noting that although the training phase only covers specific terrain parameter configurations, the dual-layer PPO still maintains a success rate significantly higher than the baseline under extreme parameters outside the training set (h=10 cm, $\theta =20^{\circ }$). This indicates that the hierarchical structure guides the policy network to extract generalized feature representations of dynamic balance, rather than overfitting to specific terrain parameters, thereby possessing a certain degree of out-of-domain generalization capability.

### 4.3. Narrow-Space Navigation Experiment

#### 4.3.1. Narrow-Space Navigation Scenario Construction

This experiment aims to verify the contribution of the global path planning module to enhancing navigation performance in complex topological spaces, as well as the impact of different low-level controllers on the execution quality of navigation commands. To eliminate the interference of differences in low-level control capabilities on the experimental conclusions, all comparative groups use narrow-space scenarios of the same difficulty level. The comparative algorithms are configured as follows:

Pure PPO (Baseline 1): Without incorporating any global path planning module, this approach relies solely on a single-layer PPO network for end-to-end navigation and balance control. The network directly receives LiDAR environmental perception data and vehicle dynamics state information, outputting steering angle, vehicle speed, and momentum wheel torque commands, and autonomously explores navigation strategies in narrow spaces through reinforcement learning.

RRT* + LQR (Baseline 2): Employs a traditional classical control method as the low-level executor. The Linear Quadratic Regulator (LQR) is designed based on the linearized bicycle model and possesses theoretical optimality near the equilibrium point; however, its reliance on the model linearization assumption leads to limited robustness under large disturbances and strongly nonlinear conditions.

RRT* + Single-layer PPO (Baseline 3): Utilizes RRT* to provide global path guidance, with a single-layer PPO network serving as the low-level executor, handling both path tracking and balance maintenance. This baseline is used to reveal the performance upper bound of non-hierarchical reinforcement learning architectures under the premise of incorporating global planning.

RRT* + Dual-layer PPO (Proposed): Utilizes RRT* to provide global path guidance, combined with the proposed dual-layer PPO architecture as the low-level executor. The upper-layer network generates the desired compensation values that comply with bicycle kinematic constraints based on route point guidance, obstacle distribution, and terrain undulations, while the lower-layer network ensures dynamic stability of the vehicle during maneuvering in confined spaces.

Evaluation metrics include Task Success Rate, Average Path Length, and Average Task Completion Time. In practical application scenarios of autonomous bicycles, narrow unstructured environments such as crowded corridors, forest trails, and complex indoor mazes impose extremely high requirements on the global planning capability and local obstacle avoidance flexibility of the algorithm. The typical characteristics of such scenarios are narrow feasible regions, dense obstacle distributions, and complex topological structures, which easily cause reinforcement learning algorithms relying solely on local perception to fall into “local minimum” traps or suffer collisions due to a lack of long-horizon planning vision.

To comprehensively evaluate the navigation performance of each algorithm under different geometric constraints and topological complexities, four representative test scenarios are constructed in the PyBullet simulation environment.

**Figure 11:**
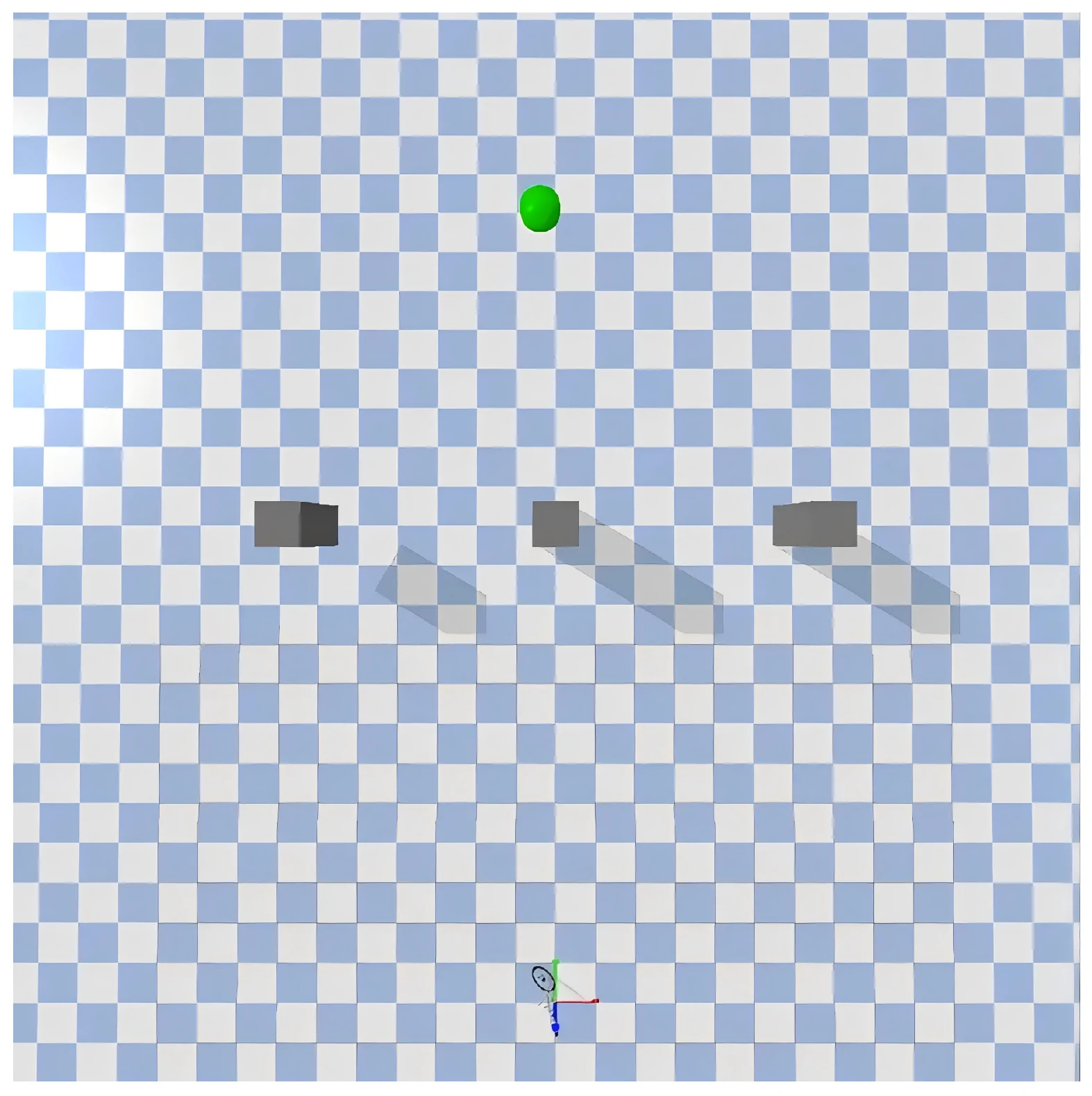
Equally spaced block scenario. The green ball indicates the target point.

Equally Spaced Block Scenario (Figure 11): Obstacle blocks are uniformly distributed at fixed intervals in an open area, forming a regular sparse obstacle distribution with relatively wide passages and no complex topological dead ends. This scenario serves as a low-difficulty navigation baseline. Its core function is to verify the reasonableness of algorithm parameter settings by providing a friendly test environment for all controllers. If an algorithm performs well in this simple scenario, then failures in more complex scenarios can be attributed to limitations of the algorithm architecture itself rather than improper parameter tuning.

**Figure 12:**
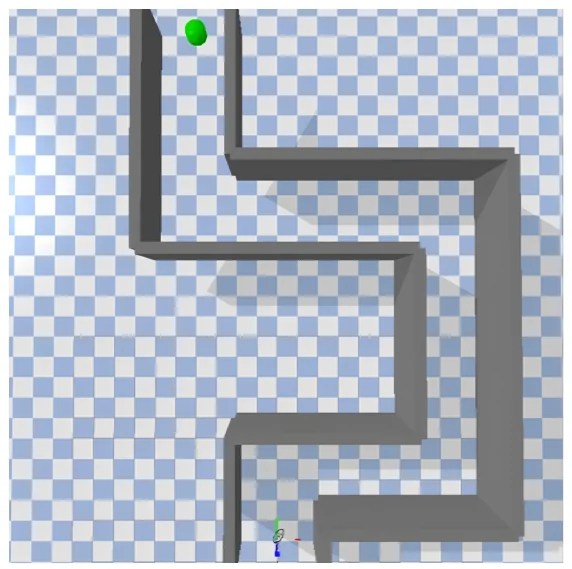
S-shaped tortuous corridor. The green ball indicates the target point.

S-Shaped Tortuous Corridor (Figure 12): Corridor width is approximately 1.5 times the vehicle body width, containing continuous sharp turns. This scenario primarily examines trajectory smoothness and continuous steering execution capability in constrained spaces, and requires the controller to have forward-looking path prediction capability to avoid collisions with corridor walls.

**Figure 13:**
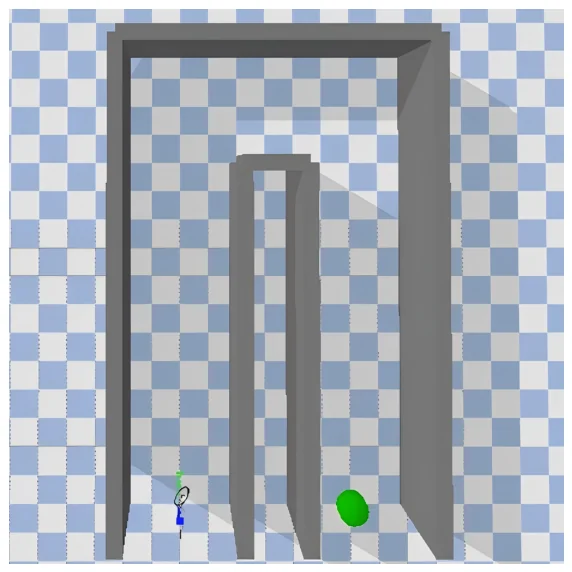
U-shaped dead end scenario. The green ball indicates the target point.

U-Shaped Dead End (Figure 13): The start and goal points are on opposite sides of a U-shaped obstacle, requiring the agent to actively execute long-sequence actions of first moving away from the target and then circumnavigating to arrive. This is a classic “local minimum” test benchmark specifically evaluating the algorithm’s ability to escape local greedy strategies and complete global topological detours.

**Figure 14:**
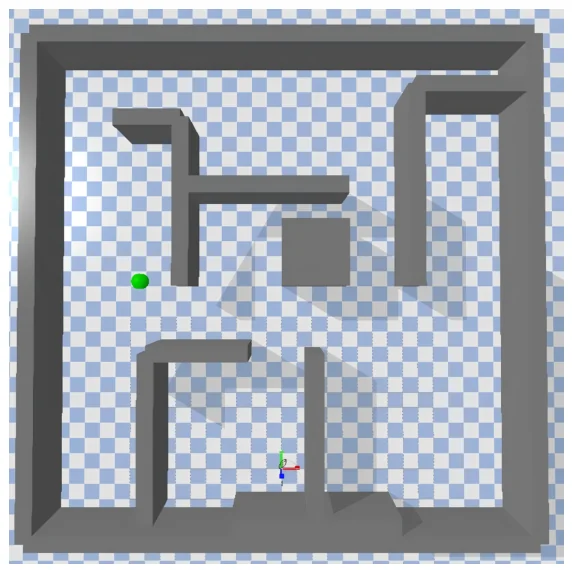
Random dense maze. The green ball indicates the target point.

Random Dense Maze (Figure 14): Multiple static obstacles are randomly generated in a 30m×30m area, forming a narrow passage network without fixed patterns. This scenario simulates unknown complex unstructured real-world environments, primarily evaluating generalization and zero-shot adaptation capability.

Each scenario is tested independently for 200 trials. Task success is defined as the bicycle successfully reaching the target point (position error <0.5 m) within the prescribed time without collision or falling.

#### 4.3.2. Narrow-Space Navigation Experimental Results and Analysis

Table 4 summarizes the statistical results of the four comparative algorithms across the four narrow-space scenarios with 200 independent trials each.

The experimental results demonstrate that the proposed RRT*-2LPPO method achieves the best navigation success rates across all four scenario types, with significant performance gaps compared to all baseline algorithms. A detailed analysis follows from several dimensions.

Verification of RRT* + LQR as a valid control baseline. RRT* + LQR achieved a 94.0% success rate in the equally spaced block scenario, close to the proposed method’s 99.5%, and its average completion time (15.24 s) is comparable to that of the proposed method (14.1 s). This confirms that the LQR control parameters designed in this study fall within a reasonable range. The sharp drop in performance of this method to 5.5% and 0% in the S-shaped corridor and U-shaped dead end scenarios, respectively, is the result of the inherent structural limitation of the equilibrium point linearization model in narrow environments, not an engineering problem that can be avoided through parameter optimization.

Necessity of global planning. Pure PPO achieved a 0% success rate in both the S-shaped tortuous corridor and U-shaped dead end scenarios, and only 24.0% in the random dense maze. Its average path length (11.0 m) was significantly shorter than those of other methods incorporating global planning. This does not reflect an advantage in path efficiency, but rather indicates that the agent prematurely terminates the task over short distances due to collisions or a loss of directional control. These results clearly show that in topologically complex narrow environments, end-to-end reinforcement learning relying solely on local perception faces a fundamental planning bottleneck: without long-horizon vision, the algorithm is unable to handle scenarios that require detour navigation.

Decisive role of low-level controller quality. In the S-shaped tortuous corridor, RRT* + LQR achieved only 5.5%, barely higher than the completely failing pure PPO baseline; the performance gain from introducing global planning is almost negligible. In the U-shaped dead end, the success rate is also 0%. The root cause lies in the inherent limitation of the LQR controller. Notably, RRT* + LQR achieved 40.5% in the random dense maze, higher than the single-layer PPO without planning, showing that global path guidance can still play a role in relatively open maze corridors. However, in scenarios with higher demands on lower-level dynamic stability, LQR’s insufficient robustness has become the bottleneck that determines the overall performance ceiling.

Performance gain of the hierarchical architecture. In the equally spaced block scenario, the success rate of RRT* + Single-layer PPO (63.5%) is already significantly lower than that of the proposed method (99.5%), showing that upon introducing global waypoint guidance, the multi-task gradient conflict of the single-layer network is immediately apparent. In the three complex scenario types, the success rates of RRT* + Single-layer PPO are 38.5%, 28.0%, and 58.5% respectively, which are improved compared to the pure PPO baseline, proving that global path guidance can effectively alleviate the sparse reward and local minimum problems. However, there remains a gap of more than 40 percentage points compared to the proposed method. In the most topologically challenging U-shaped dead end scenario, the proposed method completed all tests with a 100% success rate, whereas RRT* + Single-layer PPO and RRT* + LQR achieved only 28.0% and 0%, respectively. This powerfully proves that guaranteeing low-level dynamic stability is the decisive factor in achieving high-success-rate navigation.

In terms of path efficiency, the average path lengths of the proposed method across the four scenario types (24.2 m, 39.8 m, 30.1 m, 21.9 m) are on the same order of magnitude as those of other methods incorporating global planning, with no sign of significantly redundant detours. Furthermore, its average completion times (14.1 s, 22.8 s, 17.3 s, 12.7 s) are all superior to or on par with RRT* + Single-layer PPO, confirming the comprehensive efficiency advantage of the hierarchical architecture.

### 4.4. Qualitative Analysis of Navigation Trajectories

The previous two sections have validated the performance superiority of the proposed framework from the perspective of quantitative metrics. However, quantitative metrics alone cannot fully reveal the essential differences in navigation behavior patterns between the two architectures. Since the earlier quantitative experiments have fully demonstrated the indispensability of the global planning module (RRT*) in complex constrained spaces, this section strictly follows the principle of controlled variables and selects only RRT* + Single-layer PPO as the core comparative baseline, since it similarly possesses antecedent RRT* planning capability. This setting eliminates interference from macroscopic path planning as much as possible, allowing isolated examination of the lower-level performance difference between “dual-layer hierarchical execution” and “single-layer end-to-end execution” when dealing with complex nonholonomic dynamic constraints.

**Figure 15:**
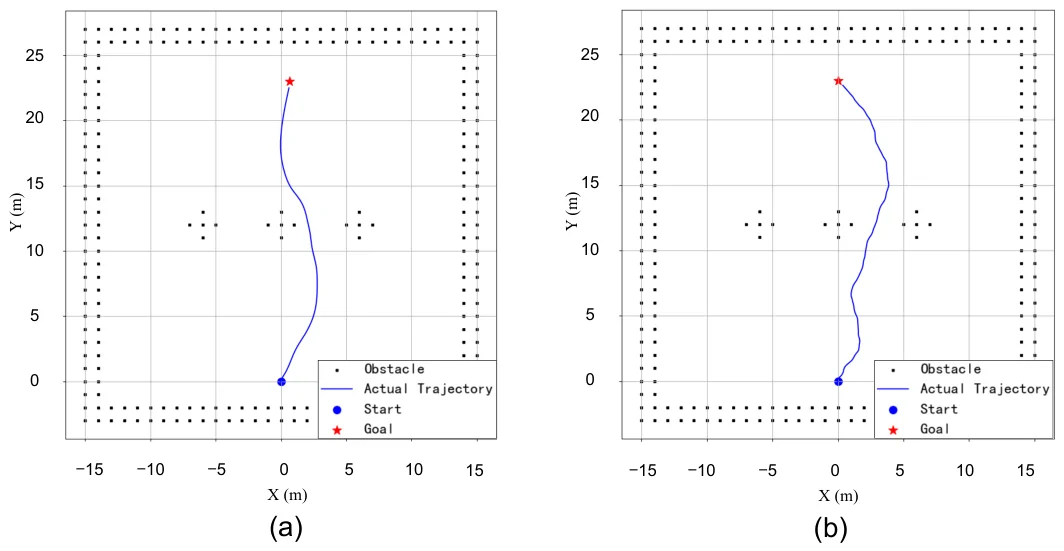
Navigation trajectory comparison in the equally spaced block scenario: (**a**) RRT*-2LPPO; (**b**) RRT* + Single-layer PPO.

**Figure 16:**
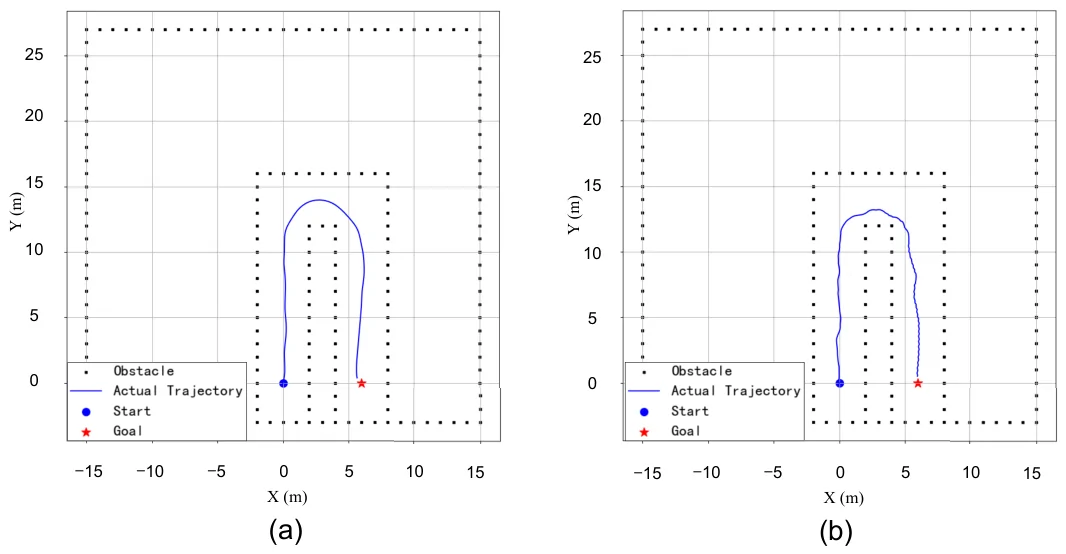
Navigation trajectory comparison in the S-shaped tortuous corridor: (**a**) RRT*-2LPPO; (**b**) RRT* + Single-layer PPO.

**Figure 17:**
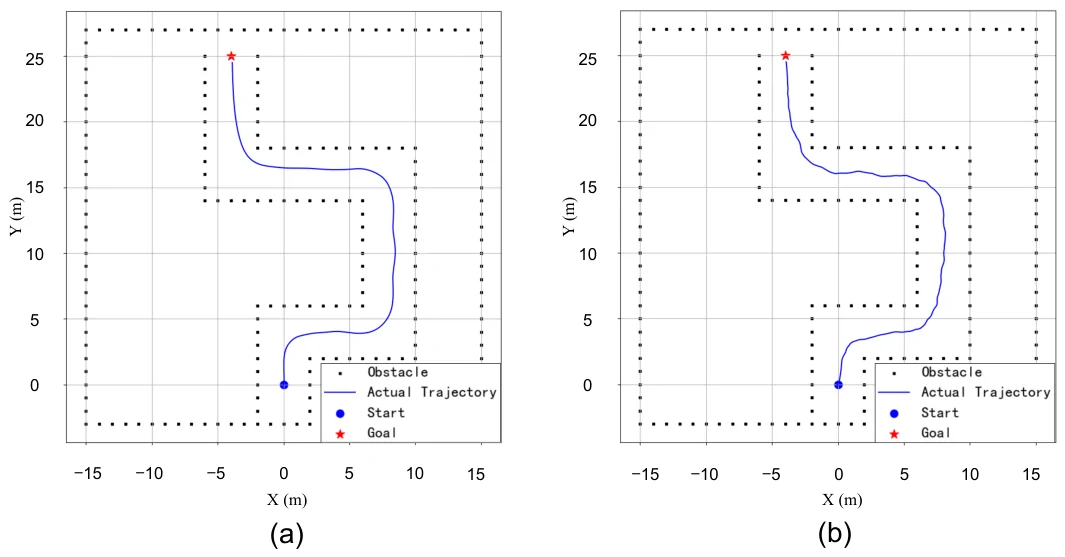
Navigation trajectory comparison in the U-shaped dead-end scenario: (**a**) RRT*-2LPPO; (**b**) RRT* + Single-layer PPO.

**Figure 18:**
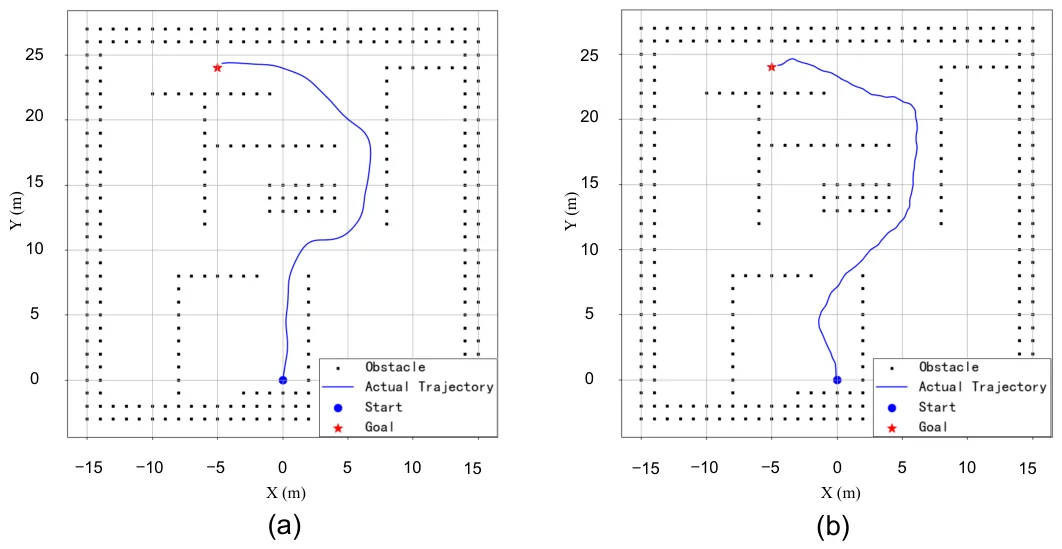
Navigation trajectory comparison in the random dense maze: (**a**) RRT*-2LPPO; (**b**) RRT* + Single-layer PPO.

Figure 15, Figure 16, Figure 17 and Figure 18 illustrate the top-view trajectory comparisons of the two methods across four types of typical scenarios, where the blue solid lines represent the actual motion paths of the bicycle’s center of mass. In each figure, sub-figure (a) shows the trajectory of the proposed RRT*-2LPPO method, and sub-figure (b) shows the trajectory of the baseline RRT* + Single-layer PPO.

High-frequency oscillation suppression and control smoothness. A cross-scenario comparison indicates that the driving trajectories of the baseline algorithm show high-frequency lateral jitter, presenting prominent “zigzag” characteristics in all scenarios. This is particularly pronounced when approaching the target in a straight line (Figure 17b) and within the long straight passages of the maze (Figure 18b). This reveals the strong coupling defect of the single-layer reinforcement learning network when simultaneously handling “macroscopic path tracking” and “fine-grained attitude balance”: to keep the vehicle upright, the network frequently outputs extreme steering commands, causing the actual trajectory to repeatedly oscillate on both sides of the reference path. In contrast, the trajectories of the proposed method (Figure 15a, Figure 16a, Figure 17a and Figure 18a) exhibit significantly superior smoothness and continuity. The upper-layer kinematic strategy outputs smooth desired commands, while the lower-layer dynamic strategy absorbs physical disturbances with high-frequency responses. This task decoupling effectively filters out high-frequency noise from the control commands.

Dynamic adaptability for large-curvature cornering. Executing large-curvature steering in constrained spaces is a core difficulty in testing the control performance of underactuated systems. In the S-shaped corridor (Figure 16) and U-shaped dead end (Figure 17) scenarios, the vehicle needs to execute continuous sharp turns to avoid dense boundary obstacles. The trajectory transitions of the baseline algorithm are rigid at the bend vertices and exit phases, causing the vehicle body to dangerously approach the obstacle boundaries and severely compressing the safety margin. More importantly, an in-depth analysis of the failure cases of the baseline algorithm reveals its fatal limitation in continuous maneuvering conditions: when facing continuous large-angle steering, the single-layer network can barely maintain balance for only one or two successive turns. As subsequent continuous steering actions accumulate, the attitude recovery lag when dealing with drastic centrifugal force changes is continuously amplified, causing the vehicle body roll angle error to persistently accumulate without effective convergence, and the vehicle ultimately loses stability and falls over. In contrast, the proposed method (Figure 16a and Figure 17a) features natural and smooth trajectory transitions, maintaining minimal lateral tracking error in the bend regions. This powerfully proves the strong robustness of the lower-level controller against severe roll disturbances.

Path fidelity under complex topologies. In the random dense maze scenario (Figure 18), the global geometric reference path contains multiple abrupt curvature changes. Although the baseline algorithm successfully finds the topological pathway with the aid of RRT*, its accumulated dynamic errors gradually amplify during the long-distance, multi-turn navigation task, resulting in irregularly distorted trajectories. The proposed method maintains high-fidelity tracking of the reference path from beginning to end. Even when navigating around the large L-shaped obstacle in the upper part of the figure, it still outputs a smooth envelope curve that complies with the vehicle’s nonholonomic constraints.

In summary, the trajectory visualization results further confirm the soundness of the proposed architecture: the global planning module provides correct topological guidance, while the dual-layer hierarchical control architecture fundamentally eliminates the command conflict between attitude balance and path tracking, endowing the system with high-quality dynamic navigation capability in complex unstructured environments.

## 5. Conclusions

To enable autonomous bicycles to travel smoothly on rugged terrain and avoid obstacles precisely in narrow constrained spaces, this paper proposes the RRT*-2LPPO framework. The architecture consists of a global planning layer and a local control layer. In the global planning layer, a two-stage global planning strategy combining the RRT* algorithm with cubic B-splines is adopted. The RRT* algorithm with asymptotic optimality performs probabilistic sampling in a large-scale configuration space, and cubic B-spline smoothing provides the agent with a global reference path satisfying $C^{2}$ continuity. This strategy solves the problem that paths generated by standard RRT in narrow terrain have discontinuous curvature and fundamentally cannot be executed by the bicycle due to nonholonomic constraints. It also provides the PPO algorithm with a reference trajectory that prevents local deadlocks, and significantly improves task success rates and path smoothness in complex constrained scenarios.

In the local control layer, the control and decision-making are split into two functionally complementary PPO networks, forming a hierarchically coupled cooperation between decision-making and execution. The upper-layer network fuses LiDAR data with vehicle body pitch angle information to perceive terrain slope changes in real time and generates motion strategies suited to rugged terrain. The lower-layer network uses the physical coupling characteristics of the momentum wheel system and the steering mechanism to maintain high-frequency attitude stability.

The experimental results demonstrate the outstanding performance and high robustness of the proposed architecture. In rugged terrain tests (continuous stairs and steep slopes), the hierarchical design achieves decoupling of high-frequency attitude stability from low-frequency trajectory tracking, enabling continuous climbing and descending on rugged terrain. The task success rate of the dual-layer PPO in rugged terrain reaches 97.7–99.4%, an improvement of more than 60 percentage points over the single-layer PPO baseline (17.6–30.4%). In the narrow-space navigation experiments, the proposed method (RRT*-2LPPO) not only achieves the highest navigation success rates in all challenging scenarios but also demonstrates excellent trajectory smoothness. In particular, the navigation success rate reaches 100% in the U-shaped dead end scenario and 92.5% in the random dense maze scenario, significantly outperforming baseline methods such as RRT* + LQR (0%) and RRT* + Single-layer PPO (28%/58.5%).

In summary, this paper proposes the RRT*-2LPPO framework by combining global path navigation with hierarchical reinforcement learning. A control scheme tailored to the structural characteristics of the bicycle is developed, enabling stable operation on rugged terrain and in narrow spaces. This provides a solution for the stable all-terrain traversal of autonomous bicycles and offers a new approach for the intelligent control design of underactuated mobile robots.

The present work remains at the simulation validation stage and has not yet addressed the Sim-to-Real transfer problem. Performance in real environments remains to be examined. Future research will attempt to transfer this system from simulation to a real physical prototype, and will also integrate semantic perception information from multi-sensor fusion into the decision-making framework. The ultimate goal is to advance the engineering application of autonomous bicycles in real-world scenarios such as urban last-mile delivery and specialized security patrols.

## Figures and Tables

**Table 1:** Symbol definitions and descriptions for the autonomous bicycle model.

Symbol	Description	Unit
$m_{b}$, $m_{\omega }$	Mass of vehicle body and momentum wheel	kg
$l_{b}$	Distance from vehicle body CoM to ground contact point *O*	m
$l_{\omega }$	Distance from momentum wheel rotation center to ground contact point *O*	m
$I_{b}$	Moment of inertia of vehicle body about contact point *O*	kg·m^2^
$I_{\omega }$	Moment of inertia of momentum wheel about its rotation axis	kg·m^2^
*g*	Gravitational acceleration	m/s^2^
*M*	Control torque output by the momentum wheel motor	N·m

**Table 2:** Simulation environment and bicycle model parameters.

Parameter	Value	Unit
Simulation time step	0.04	s
Gravitational acceleration	−9.8	m/s^2^
Ground friction coefficient	0.6	–
Total bicycle mass	15.2	kg
Momentum wheel mass	0.5	kg
Momentum wheel motor no-load speed	3000	RPM
Rear wheel motor no-load speed	2000	RPM
LiDAR sensor frequency	30	Hz

**Table 3:** Performance comparison of different control architectures on rugged terrain scenarios.

Algorithm	Scenario Type	Difficulty	Success Rate (%)	Avg. Time (s)
Single-layer PPO	Continuous stairs	h=5 cm	30.4	13.3
(Non-hierarchical)	Continuous stairs	h=10 cm	17.6	14.2
	Steep slope	$\theta =10^{\circ }$	59.5	12.9
	Steep slope	$\theta =15^{\circ }$	31.2	13.1
	Steep slope	$\theta =20^{\circ }$	16.2	13.2
Dual-layer PPO	Continuous stairs	h=5 cm	99.4	14.3
(Proposed)	Continuous stairs	h=10 cm	97.7	14.6
	Steep slope	$\theta =10^{\circ }$	94.8	14.8
	Steep slope	$\theta =15^{\circ }$	85.5	15.5
	Steep slope	$\theta =20^{\circ }$	69.4	15.2

**Table 4:** Navigation performance comparison of different algorithms in narrow-space scenarios.

Algorithm	Scenario	Success Rate (%)	Avg. Path Length (m)	Avg. Time (s)
Pure PPO	Block scene	93.5	22.4	13.0
(no global plan)	S-shaped corr.	0	– *	– *
	U-dead end	0	– *	– *
	Random maze	24	11.0	6.5
RRT* +	Block scene	63.5	24.1	14.6
Single-layer PPO	U-dead end	28	29.3	18.2
	Random maze	58.5	20.6	13.5
RRT* + LQR	Block scene	94	24.2	15.24
	S-shaped corr.	5.5	37.3	25.5
	U-dead end	0	– *	– *
	Random maze	40.5	14.4	8.7
RRT* + Dual-layer PPO	Block scene	99.5	24.2	14.1
(Proposed)	S-shaped corr.	99.5	39.8	22.8
	U-dead end	100	30.1	17.3
	Random maze	92.5	21.9	12.7

* Not applicable: the algorithm achieved a 0% success rate in this scenario, meaning no trial reached the goal point; path length and completion time are therefore undefined and not recorded.

## Data Availability

The code will be made public after the recruitment process.
